# Effectiveness of a blended school-based mindfulness program for the prevention of co-rumination and internalizing problems in Dutch secondary school girls: a cluster randomized controlled trial

**DOI:** 10.1186/s13063-023-07885-x

**Published:** 2024-01-12

**Authors:** Patricia Vuijk, Kim Bul, Marieke Buil, Marloes Rauws, Keshia Curie, Charlotte Amesz, Ron Weerheijm, Heleen Riper

**Affiliations:** 1 Research Centre Innovations in Care, Rotterdam University of Applied Sciences, Rotterdam, the Netherlands; 2 Research Centre Urban Talent, Rotterdam University of Applied Sciences, Rotterdam, the Netherlands; 3 Institute for Health and Wellbeing, Centre for Intelligent Healthcare, Coventry University, Coventry, UK; 4 Department of Clinical, Neuro- and Developmental Psychology, section Developmental Psychology, Vrije Universiteit Amsterdam, Amsterdam, the Netherlands, Vrije Universiteit Amsterdam, Amsterdam, the Netherlands; 5 Department of Clinical, Neuro- and Developmental Psychology, Section Clinical Psychology, Vrije Universiteit Amsterdam, Amsterdam, the Netherlands

**Keywords:** Adolescence, Girls, Co-rumination, Internalizing problems, Prevention, Mindfulness, Cluster Randomized Controlled Ttrial

## Abstract

**Background:**

A growing body of literature indicates that adolescent girls who talk with close friends about interpersonal problems or worries in an excessive, speculative way, and with an intense focus on distress (i.e., *co-rumination*) are at heightened risk for developing internalizing symptoms and disorders as well as reduced friendship quality. However, to date, there are no prevention programs available that target high levels of co-rumination between adolescent girls. As such, we developed the blended school-based mindfulness prevention program Happy Friends, Positive Minds (HFPM) that targets co-rumination at the dyadic level, i.e., between two close female friends. The aim of this trial is to evaluate the effectiveness of HFPM to reduce co-rumination and internalizing problems and to enhance wellbeing and social-emotional behavior in Dutch adolescent girls.

**Methods:**

A cluster Randomized Controlled Trial (cRCT) will be conducted to evaluate HFPM effectiveness. We will recruit 160 female friendship dyads (*n* = 320 girls) aged 13 to 15 years who will be characterized by high levels of self-reported co-rumination. The cRCT has two arms: (1) an intervention condition in which 160 girls (80 friendship dyads) will receive the 14-week HFPM program in two consecutive cohorts (cohort 1 in academic year 2023/2024 and cohort 2 in academic year 2024/2025, and (2) a control condition in which 160 girls (80 dyads) will receive care-as-usual (CAU) in two consecutive cohorts (cohort 1 in academic year 2023/2024 and cohort 2 in academic year 2024/2025). Data will be collected at baseline (T0), during the program (T1;T2; T3), immediately after the program (T4), and at 1-year follow-up (T5). Participant-level self-reported risk for (early onset) depression and anxiety, self-reported and observed co-rumination, self- and friend-reported friendship quality, self-reported positive and negative affect, self-reported interpersonal responses to positive affect, and self-reported anhedonia symptoms will be the outcome variables.

**Discussion:**

This study will provide insights into the short-term and long-term effects of the HFPM program on girls’ internalizing problems, wellbeing, and social-emotional behavior.

**Trial registration:**

International Standard Randomized Controlled Trials, identifier: ISRCTN54246670. Registered on 27 February 2023.

**Supplementary Information:**

The online version contains supplementary material available at 10.1186/s13063-023-07885-x.

## Background

Adolescence is characterized by a profound shift in the nature and significance of relationships. During this challenging developmental phase, adolescents start spending more time with friends and become increasingly independent from their parents [1]. Striving toward greater autonomy from parents and establishing positive, stable relationships with friends potentially creates stressful challenges and intense, complex pleasant and unpleasant emotions for adolescents. Other challenges during adolescence include managing increased school-related expectations and responsibilities, dealing with dilemmas on social media, developing romantic relationships, managing greater financial responsibilities, and balancing these new responsibilities altogether [2]. Related intense pleasant and unpleasant emotions are becoming increasingly important topics of adolescents’ conversations with friends [3]. Navigating these developmental challenges, while disclosing and interacting about intense emotions, result in more dependency on friends who become central sources of support during this developmental period [4].

To be able to effectively respond to the stressors related to adolescence, emotions must be modified regularly, a process known as emotion regulation [5]. Emotion regulation becomes increasingly important for adolescents’ mental health [6]. Particularly during adolescence, youth increasingly rely on *interpersonal emotion regulation* [7]. Interpersonal emotion regulation encompasses seeking interpersonal interaction to regulate one’s own emotions as well as deliberately influencing other people’s emotions [5]. One important interpersonal emotion regulation strategy during adolescence is *co-rumination* [8]. Co-rumination refers to excessively discussing personal problems within a dyadic relationship and is characterized by frequently discussing problems, discussing the same problem repeatedly, mutual encouragement of discussing problems, speculating about problems, and focusing on unpleasant feelings and emotions [9].

Co-rumination has been conceptualized serving an *emotion regulatory function* [7, 8] because its use is intended to regulate or modify distressing unpleasant emotions or distressing emotional experiences [10]. Because co-rumination involves high levels of self-disclosure (i.e., sharing one’s thoughts and emotions), and adolescents carefully select peers who are skilled in assisting their emotion regulation processes, co-rumination often manifests itself in high-quality, close dyadic friendships [11]. Prior research has demonstrated that co-rumination leads to greater feelings of closeness and better relationship quality [9, 11, 12]. These benefits are most likely caused by the presence of self-disclosure, which enhances intimacy and increases attraction within close dyadic friendships [13].

However, although the transition to adolescence marks a period of establishing supportive and close friendships, early adolescence is also a vulnerable period for adolescents’ mental health development, characterized by an explosive increase in the onset and escalation of internalizing problems [14]. Despite the benefits of disclosure within close friendships, certain aspects of co-rumination processes between friends can unintentionally cause high levels of emotional distress. Especially, high levels of excessive, pervasive co-rumination about negative content (i.e., unpleasant emotions, distressing experiences) within friendships have been linked to elevated internalizing symptoms [15]. Furthermore, co-rumination has been found to predict clinical depression [16], as well as the (first) onset, severity, and duration of future depression [17].

Longitudinal evidence has indicated that gender plays a critical role in the development of excessive co-ruminating interaction patterns and related internalizing problems. Girls have been found to be especially at risk. Girls consistently are found to disclose emotionally charged and complex interpersonal problems and worries with their friends more than boys [18], discuss interpersonal problems with friends more often compared to boys [3] and report more distress about interpersonal problems and worries than their male counterparts [19]. Moreover, girls engage in more perspective taking, which is associated with better relationship quality but also with more empathic distress [20]. Indeed, a breadth of research demonstrated that late childhood female friendships are characterized by higher rates of co-rumination compared to boys, a difference that becomes even more pronounced during adolescence [11, 21–23].

These patterns fit with the developmental psychology perspective that interpersonal communication and disclosure is more central to girls’ than to boys’ identities [24]. More specifically, during adolescence, girls value emotional attachment within their friendships more than boys [24]. In the period of early adolescence, girls—in their search for their identity—are therefore more likely than boys to form friendships in which mutual emotional connection is present and can intensify. When girls respond empathically to each other’s pleasant and unpleasant emotions, bonding is created that makes friendships attractive to girls [24]. Scientific research confirms this pattern: not only might co-rumination lead to more emotional closeness, attraction, and intimacy; conversely, more closeness, attraction, and intimacy also predict higher levels of co-rumination in adolescents [9, 11, 12, 25]. So, it seems that the better relationship quality girls experience in a friendship relationship, the more they disclose about their problems, concerns, and emotions with close friends.

However, co-rumination within close, dyadic female friendships can cause elevated risks for internalizing symptoms and disorders by functioning as an *ineffective dyadic emotion regulation strategy*. In particular, when excessive, passive and repeated sharing of unpleasant emotions and unpleasant topics has become the ‘default mode’ of communication, concerns and problems appear larger, creating a sense that there are no solutions. Girls who become fixated on unpleasant topics have difficulty shifting their attention: unpleasant topics actively dominate working memory [6]. It can be concluded that co-rumination is an interpersonal emotion regulation process that may be of use in explaining why seemingly supportive discussions between adolescent girls lead to increased distress and elevated risk for internalizing problems, as girls perseverate on problems with their friends, whereby one or both girls actively focusing repetitively and intensely on negative emotions and distress with another girl [9].

There are several mechanisms by which a perseveration on discussing negative affect influences the effectiveness of emotion regulation in daily life and confers risk for the development and exacerbation of internalizing problems. First, co-rumination increases negative affect (e.g., anger, sadness, or nervousness) in individuals during co-rumination discussions within dyads, particularly when there is no attempt to move toward distraction or problem solving [26]. Second, co-rumination contributes to maintenance of negative affect in individuals immediately following co-rumination discussions [27] and fosters the development of individual ruminative tendencies outside of the interactions [12, 25, 28], a transdiagnostic factor for anxiety and depression [29]. Third, co-rumination has also been found to negatively impact friendships. For instance, Mackenzie and colleagues [30] demonstrated that co-rumination suppressed the beneficial effect of seeking support from friends. Diminished friendship quality and interpersonal difficulties are predictive of depression and anxiety [31]. Together, these results provide strong support that co-rumination may be reinforced by social benefits (e.g., intimacy, validation) rather than by affective relief.

Co-rumination not only increases individual risks for the development of depression and anxiety in girls, but also poses a risk for mutual depression and anxiety *contagion* [23, 32]. Depression and anxiety contagion occurs when friends’ depressive and anxiety symptoms predict increases in adolescents’ own depressive and anxiety symptoms over time and vice versa [23]. Previous studies identified three ways in which co-rumination might mediate contagion effects. First, adolescents can strongly experience ‘empathetic distress’ in response to their friends’ problems or worries, meaning that they share in their friends’ distress in ways that they are taking on the distress as their own [20, 33]. More specifically, adolescents who repeatedly and excessively are exposed to friends expressing, speculating, and rehashing on personal distress through co-rumination may become distressed themselves. Second, adolescents may become distressed as a result of co-rumination with friends with internalizing symptoms because those friends might offer particularly pessimistic perspectives, because they redirect interactions about worries and problems to focus on the self (e.g., conversational self-focus; [34]) or because of high reassurance seeking [32]. This suggests that contagion of depressive and anxiety symptoms within friendships may not only occur because of more exposure to the friends’ distress, but also may be due to adolescents’ negative emotions (e.g., irritation, helplessness) in response to friends’ behavior. Finally, time spent on excessive co-rumination hampers adolescents spending time on more positive activities that offset negative affect.

These results have important clinical implications, especially for girls. Co-rumination serves as a socially rewarding process within girls’ friendships, which may perpetuate or maintain the tendency to co-ruminate. Since peers are an important source of social support during adolescence, interventions are warranted that specifically provide opportunities to teach girls better emotion regulation skills thereby reducing the particular risk for excessive co-rumination and early onset of depression and anxiety. However, surprisingly, to date, there are no prevention programs available that target co-rumination in young adolescent girls.

The described patterns have several important clinical implications for girls who are using dwelling on negative affect as dominant emotion regulation strategy within their dyadic conversations with their best or close same-sex friends:

1. First, because co-rumination is strongly associated with feelings of closeness and support within girls’ friendships, it is important to target positive friendship quality as a starting point for intervention [34].
2. Second, girls should be encouraged to become aware of the way in which they communicate with close friends and of the potential risks of excessive problem talk [35].
3. Third, girls may benefit from explicit instructions on becoming more aware of their emotional states and then learning and practicing effective ways of managing and communicating about intense emotions [36].
4. A fourth important goal for interventions is to support girls in exploring ways of balancing excessive, negatively focused and speculative problem talk with positive activities and positive topics. This will help girls to prevent a perseverative focus on negative affect and to revisit joys with friends in their everyday life [34].
5. A fifth important goal for interventions is to support ways to experience positive rumination and empathetic joy, i.e., sharing in a friend’s positive emotions [20, 37].
6. A sixth goal is to teach girls adaptive emotion regulation strategies to help set healthy boundaries between friends’ negative emotions and their own [36].
7. Finally, an important objective of prevention is to teach girls emotional competences that enable them to set healthy boundaries between the unpleasant emotions of friends and their own [36].

Because secondary schools play a crucial role in the lives of adolescent girls and because of their wide and diverse reach, they provide a unique setting for identifying high-risk friendships, for preventing mental health problems and for promoting mental health prevention strategies [38]. However, although extensive research has emphasized the negative implications of co-rumination, thereby linking high co-rumination to higher levels of internalizing symptoms and internalizing disorders [17] and the fact that only approximately half of adolescents with a mental disorder receive treatment [39], school-based prevention programs for high-risk co-ruminating girls addressing the abovementioned clinical implications are not available yet.

In this study protocol, the Happy Friends, Positive Minds prevention program is introduced. This program is a mindfulness-based prevention program that aims to prevent excessive co-rumination in girls from age 13 to 15 years old. In this program, mindfulness is operationalized as the natural and trainable ability to use qualities such as attentiveness, kindness, curiosity, and responsiveness to bring awareness to both internal (bodily sensations, emotions, thoughts, behaviors) and external (stressors, friendships) experiences, without judgment and with deliberate attention in the here and now [40, 41], enabling people to respond more skillfully and with greater insight [42]. Participants in mindfulness-based programs learn the ability to respond by responding more consciously and skillfully [43].

*Mindfulness training* may be particularly beneficial for girls who engage excessively together in repetitive and negative interactions given the emphasis on the cultivation of developing positive aspects of the self, developing positive relationships with others, caring for others, cultivating present-moment awareness of one’s experience, practicing appreciation and gratitude, and meta-awareness of the dynamics between thoughts, emotions, body sensations, and impulses [44, 45]. Consequently, mindfulness training may not only reduce current co-rumination and internalizing symptoms, but also may help lower the risk of internalizing disorder onset and finally may also stimulate resilience and flourishing in young girls [46].

Mindfulness-based training programs are informed by science, education, training and supervision, and contemplational practices. These programs teach foundational skills of self-regulation and attention and are non-stigmatizing [47]. There is promising evidence from randomized controlled trials that mindfulness-based programs reduce symptoms of depression, anxiety, and stress in adolescents [48–50], and especially girls showing benefits in emotional regulation [51], anxiety [51], and positive affect [52]. Evidence-based mindfulness programs are commonly described as educational or skills training programs, rather than forms of psychotherapy, but are often used to reduce psychological symptoms and stress in (sub)clinical populations. However, the literature on harmful effects of mindfulness programs for adolescents is sparse [53].

Digital technologies, including smartphone apps, provide an important avenue to increase implementation fidelity to evidence-based interventions for adolescents with mental health problems [44]. Apps may help overcome barriers associated with traditional psychotherapeutic or psychiatric treatment, such as social stigma [54]. In 2019, approximately 84% of 13- to 18-year-olds owned a smartphone [55] and 72% of the adolescents would use an app to address their mental health problem and almost 32% prefers this above face-to-face support [56]. As such, we developed the App yourself Happy app aimed at supporting excessive co-ruminating girls to integrate these mindfulness skills in their daily lives [57, 58].

This prevention program is designed to train emotion regulation skills within the supportive close friendship context by facilitating dyadic as well as individual experiential learning (e.g., learning by reflection on experiences during the practices). This program was developed between 2020 and 2023 using the Intervention Mapping Approach for planning health promotion programs [59]. The program comprises of 14 guided, weekly online lessons with mindfulness practices and psychoeducation, guiding the dyadic use of the eMental health application App yourself Happy [58]. The goal of this program is to train 160 Dutch (80 dyads) high-risk girls between ages 13 and 15 to shift dyadic maladaptive emotion regulation (ER) patterns to more adaptive ER strategies within their dyadic interactions, while continuing to reap the benefits of their close, intimate friendships and exploring healthy, new alternatives for excessive co-rumination.

The objective of the HFPM cluster Randomized Controlled Trial (cRCT) is to evaluate the effectiveness of the prevention program HFPM, delivered by experienced mindfulness health professionals, compared with care-as-usual (CAU) and to unveil mechanisms of change. Given that, to date, no other interventions are available that target excessive co-rumination between adolescent girls, girls in the control condition will receive CUA to test whether our prevention program has additional value above what is usually done in their routine care. The main research questions are:

1. What is the effectiveness of the Happy Friends, Positive Minds mindfulness-based prevention program on levels of co-rumination and risk for early-onset depression and anxiety in Dutch adolescent girls from age 13 to 15 years old?
2. What is the effectiveness of the Happy Friends, Positive Minds mindfulness-based prevention program on friendship quality, levels of self-reported positive and negative affect, interpersonal reactivity to personal distress, interpersonal responses to positive affect, anhedonic symptoms, mastery, and health care use in Dutch adolescent girls from age 13 to 15 years old?

### Main study aim, research question, and hypotheses

The main study aim of the HFPM cRCT is to evaluate the effectiveness of the school-based mindfulness program HFPM on self-reported mental health outcomes in a sample of 320 Dutch girls aged between 13 and 15 years. The overall research question is: To what extent does the app-based mindfulness prevention program HFPM impact mental health and wellbeing in Dutch 13-to-15-year-old girls?

The hypotheses are:

1. Girls in the intervention group will have a greater reduction in co-rumination about distress and difficult emotions and feelings, (and thereby) internalizing symptoms and negative affect during the intervention period, immediately after the intervention period and after 1-year follow-up, relative to girls in the control condition.
2. Girls in the intervention group will have a later onset of depressive symptoms or a later onset of depressive disorder and less dyadic depression contagion, immediately after the intervention period and after 1-year follow-up, relative to girls in the control condition.
3. Girls in the intervention group will demonstrate less anxiety, problem talk, a later onset of anxiety symptoms or a later onset of anxiety disorder and less dyadic anxiety contagion during the intervention period, immediately after the intervention period and after 1-year follow-up, relative to girls in the control condition.
4. Girls in the intervention group will experience better friendship quality, higher levels of positive affect and higher levels of interpersonal responses to positive affect of the dyad friend, during the intervention period, immediately after the intervention period and after 1-year follow-up, relative to girls in the control condition.
5. The hypothesized intervention effects on co-rumination will be mediated by the development of mindfulness skills, emotion regulation skills, and problem-solving skills during the intervention period, immediately after the intervention period and after 1-year follow-up.
6. The hypothesized intervention effects on co-rumination will be moderated by self-control: girls with more developed self-control skills will demonstrate greater intervention effects immediately after the intervention period and after 1-year follow-up, relative to girls in the control condition.
7. Girls in the intervention group will experience less anhedonic symptoms, will experience greater feelings of mastery, and will show less health care use during the intervention period, immediately after the intervention period and after 1-year follow-up, relative to girls in the control condition.
8. Girls in the intervention group will demonstrate a change in topics discussed: girls will demonstrate less problem talk about interpersonal problems and shorter periods of interpersonal problem talk immediately after the intervention period and after 1-year follow-up, relative to girls in the control condition.

### Trial design

The present study is a parallel group superiority trial design (two cohorts, see Timeline of the interventions and measures) comparing the HFPM intervention arm with a CAU control arm in a two-armed cluster randomized controlled trial. Dyads will be randomly allocated to either the HFPM program or the CAU control condition, with an equal allocation ratio (see also “Randomization, blinding, and treatment allocation”). Blinding for the intervention is not possible.

## Methods/design

### Study design

This paper used the SPIRIT reporting guidelines [60]. The aim of the HFPM cRCT is to evaluate the effectiveness of the school-based targeted mindfulness and psychoeducation blended prevention program HFPM, delivered by trained and experienced mindfulness health professionals, compared with CAU in which girls will receive care-as-usual. The HFPM is a cRCT with two arms: (1) an intervention condition in which 80 girls’ friendship dyads will receive the HFPM program, and (2) a control condition in which 80 girls’ friendship dyads will receive CAU. To prevent contamination across the two trial arms, friendship dyads will be the unit of randomization (see “Randomization, blinding, and treatment allocation”).

### Timeline of the intervention and measures

**Fig. 1:**
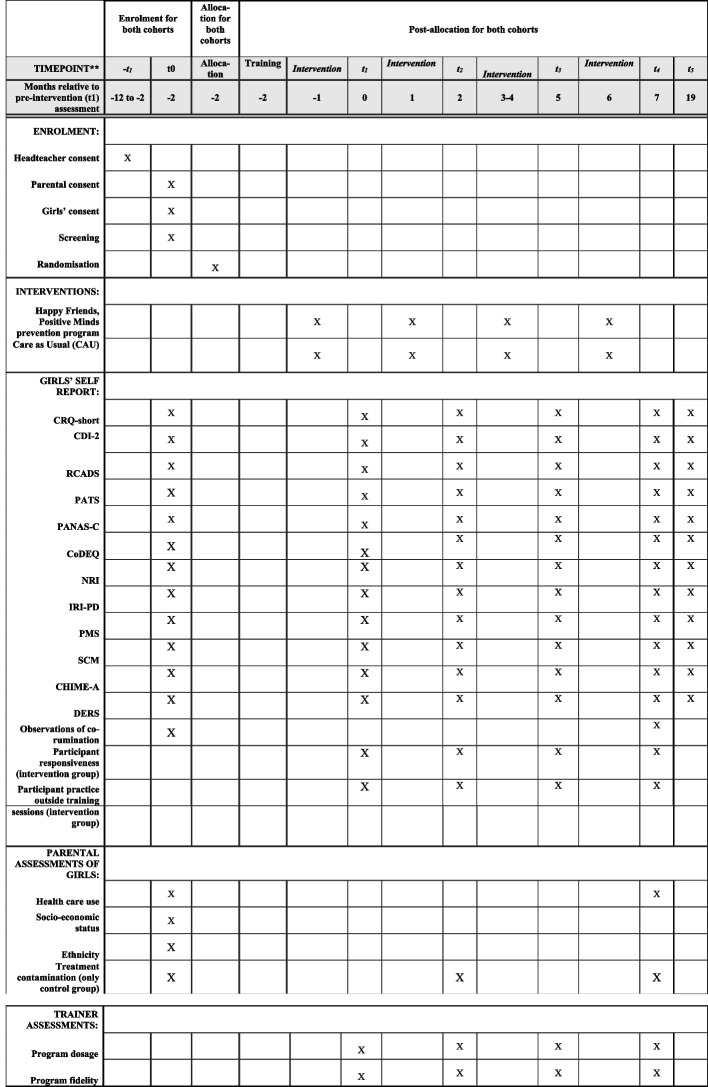
Timeline of the intervention and measure

The 14-week HFPM program will be delivered in two cohorts (cohort 1: academic year 2023–2024; cohort 2: academic year 2024–2025) in four phases between February 2024 and May 2025 for both cohorts. The T0 baseline measurements for both cohorts will take place from October 2023 (cohort 1) and from October 2024 (cohort 2), followed by T1 measurements (from February 2024 for cohort 1 and from December 2024 for cohort 2), T2 measurements (from April 2024 for cohort 1 and from February 2025 for cohort 2), T3 measurements (from June 2024 for cohort 1 and from April 2025 for Cohort 2), T4 post-intervention measurements (from September 2024 for cohort 1 and from July 2025 for cohort 2), and a 1-year long-term follow-up measurement (T5) from July 2025 to 2026 (see Fig. 1).

### Setting

The study will take place at secondary schools of Koers VO (Rotterdam, the Netherlands).

These schools will be broadly representative of Dutch secondary schools with respect to the type of school (urban/rural, large/small and high/middle/low SES of the school).

### Recruitment

School recruitment will be conducted and finalized in the academic years 2023/2024 and 2024/2025 and started in December 2022 with a presentation about the study and the prevention program by the Principal Investigator (PI) for mental health professionals across the 82 secondary Koers VO schools (i.e., a collaborative school network). When schools consent to participate, the teacher-mentors will inform parents/main caregivers via email about school participation in the study and upcoming study procedures, such as the classroom presentations and the informed consent procedures.

Schools who express their interest in the trial will be visited between May 2023 and September 2024 by two trained PhD students. When schools consent to participate, all girls of grades 8 and 9 for both cohorts will be visited between August 2023 and December 2024 by the PhD students or trained research assistants for classroom presentations about the aim of the study, the screening procedure, the HFPM training program, the randomization procedure, and the measures and will be provided the opportunity to ask questions. The classroom meetings are informative, they are not mandatory. The teacher-mentors will inform the girls in advance that a class meeting is planned at which the PhD student or the trained research assistants will present information about the study. Girls can indicate to the teacher-mentors in advance if they do not want to be present at the classroom meeting and this choice is accepted without having to give a reason for non-participation. In the classroom meeting, girls will be explained that they are allowed to participate when we receive the informed consent forms of both girls of a dyad and the parents/caregivers of both dyads, and that it will be possible to form a dyad with a girl of another 8th or 9th grade class within the same school. Participation in more than one dyad will not be allowed. Girls will also not be allowed to form dyads during the classroom visits, and it will be explained that the teacher-mentor will be available for support when the girls experience challenges during dyad formation.

After the classroom visits, the teacher-mentors of all 8th and 9th grade classes will send information letters and digital informed consent forms to all girls and their parents/ main caretakers via email (send by teacher-mentors), with a gentle reminder after 2 weeks (email send by teacher-mentors). When there is no response from the girls, or when they explicitly choose not to participate, this choice is accepted without further inquiry. Parents/main caretakers of incomplete dyads will be contacted by telephone by the PhD students and the research assistants and will receive a reminder-telephone call after 2 weeks. All researchers who will obtain informed consent are having a recent BROK certificate (i.e., Basic course on Regulations and Organisation for clinical investigators).

To be eligible for inclusion, at least one of the two girls of one friendship dyad, or both girls, should have a score of at least one standard deviation above the mean co-rumination screening score on the Co-rumination Questionnaire-Short [21].

### Sample screening and cRCT

We will include 160 high-risk friendship dyads who are primarily characterized by high levels of co-rumination within their daily interaction patterns. “High risk” is defined on the dyadic level and implies that at least one of the two girls of one friendship dyad, or both girls, should have a score of at least one standard deviation above the mean co-rumination screening score (see below). Therefore, approximately 1000 girls of approximately 100 8th and 9th classes of Koers VO schools with written informed consent will be screened on maladaptive co-rumination through the Co-rumination Questionnaire-Short [21]. All girls will receive an email with a secured link to the online questionnaire and a request to complete the questionnaire within 1 week. A first reminder will be sent after 1 week, a second reminder after 2 weeks and finally, the research team will contact the girls by phone with a final prompt to complete the questionnaire.

After completing the screening procedure, all dyads with at least one of the two girls with a score of at least one standard deviation above the overall mean co-rumination screening score will be eligible for inclusion in the cRCT. Finally, 160 Dutch girls from age 13 to 15 years old with high levels of maladaptive co-rumination and their good or best friend from age 13 to 15 years old within the same secondary school (*n* = 320 girls; 160 dyads) will be included in the cRCT. In anticipation of 20% dropout, 16 dyads will be placed on a waiting list.

### In- and exclusion criteria

To be eligible for inclusion, a *participant/girl* must meet the following five inclusion criteria:

1. Aged 13 to age 15 years.
2. Visiting a secondary school of Koers VO (i.e., collaborative school network).
3. Attending 8th or 9th grade of mainstream secondary education in academic year 2023/2024 or 2024/2025.
4. Being a cisgender woman or being a transgender woman.
5. Having a good or best friend (*being a cisgender woman or being a transgender woman) at the same school.

To be eligible for inclusion, a *dyad/girls* must meet the following inclusion criteria:

1. By lack of an official cut-off score for high co-rumination levels, we will base our inclusion on a distribution-based technique. That is, we will include friendship dyads of which at least one of the two girls, or both girls, have a score of at least one standard deviation above the mean co-rumination screening score on the Co-rumination Questionnaire-Short [21]. This will result in a sample of the 16% highest scoring girls on co-rumination. Note that a distribution-based technique is a common-used technique to select a high-risk population of clinical significance in the absence of meaningful clinical cut-offs [61].

Exclusion criteria at the *school* level (to mitigate any risk of difficulties in trial implementation):

1. Not having a headteacher in academic year 2023/2024 or 2024/2025.
2. Judged as “inadequate” during the most recent school inspection by the Dutch Inspectorate of Education.
3. Implementing another mindfulness-based intervention in academic year 2023/2024 or 2024/2025.

Exclusion criterium at the *individual* level:

- Following and/or participating in an individual or group-based mindfulness-based training in academic year 2023/2024 or 2024/2025.

### Randomization, blinding, and treatment allocation

#### Sequence generation

Girls will be randomly assigned in a 1:1 ratio to the intervention group (80 dyads; *n* = 160 girls) or to the control group (CAU; 80 dyads; *n* = 160 girls) by an independent researcher, using CASTOR Electronic Data Capture (EDC), a web-based electronic case record form and randomization program that is compatible with the GCP guidelines.

Ideally, randomization takes place on 1 day, after the screening phase has been completed. However, when this is not possible (e.g., due to time limitations), we will follow the following procedure:

We will randomize dyads in batches of 50 dyads (100 girls). For the first batch, the mean and standard deviation (*SD*) will be computed and dyads in which one or both girls score 1 *SD* above the mean will be included in the study and randomly assigned to the treatment or control condition via a block randomization procedure (i.e., 50% of dyads assigned to the intervention arm and 50% assigned to the control arm). Next, the next 50 dyads (batch 2) will be screened, and a new mean and *SD* will be calculated for the group in total (i.e., 100 dyads). Then, dyads of batch 2 who adhere to the inclusion criteria are included and randomly assigned to the control or intervention condition. We proceed with this process until 1000 girls are screened and 160 dyads are included in the study.

#### Concealment mechanism

Intervention allocation will be concealed by using a web-based application with a computer-generated list that will not be disclosed. Participants and researchers are aware of allocation.

#### Implementation

After obtaining informed consent, dyads will be randomly allocated to one of the two intervention arms by the independent researcher using Castor EDC.

### Assignment of intervention: blinding

#### Who will be blinded

Due to the organizational structure of the prevention program, students, parents, school, and research staff will not be blind to treatment allocation.

#### Procedure for unblinding if needed

The trial design is open label so unblinding will not occur.

### Sample size calculation

Multi-level models are used with individuals nested in dyads. With a clinical relevant effect size, that is, a difference of 1 *SD* (i.e., Cohen’s *d* = 1) between the control and intervention group where high-risk girls (*M* + 1 *SD*) score on average similar to non-high-risk girls on co-rumination (*M*) after the intervention, and a small to moderate effect association between co-rumination and internalizing problems (Cohen’s *d* = 0.50; [15]), which will result in a possible reduction of depressive feelings with 0.50 *SD*, and an ICC of depressive feelings within dyads of 0.20, 80 dyads (40 control, 40 intervention) will be required (Optimal Design; [62]). However, we will aim to oversample to a maximum of 160 dyads in order to identify smaller effects (Cohen’s *d* = 0.35) that are potentially relevant for theory development, as well as our hypothesized mediating and moderating factors as described in the “Data analysis plan” section.

### HFPM app-based mindfulness training program

The HFPM school-based mindfulness prevention program [57, 58] aims to teach emotion regulation and mindfulness skills on *the dyadic level* that support resilience and the positive qualities of girls’ dyadic friendships with the aim to prevent excessive co-rumination and (early onset of) internalizing symptoms (e.g., depression, anxiety). Mindfulness will be operationalized within this program as a natural and trainable capacity to bring *awareness* to both inner (e.g., body sensations, feelings, thoughts) and outer (e.g., friendships, stressors) experiences with qualities of attention, kindness, curiosity, and responsiveness, without judgment and with being in the present moment in a sustained and intentional way [41].

The App yourself Happy app for excessive co-ruminating early adolescent female friendship dyads [58] is the backbone of the prevention program and primarily aimed at supporting girls getting out of “the autopilot of negative reactivity.” Daily working with the app is supported by 14 manualized weekly online training sessions via Teams (30–50 min each; during school hours, i.e., mentor or free homework hours; girls will receive Teams-meeting links via email), aimed to teach girls to implement the mindfulness-based principles of the app in their daily lives and throughout their friendship activities and interactions. The app and the mindfulness sessions will be using an integrative combination of behavioral activation, mood monitoring, journaling, short guided and dyadic meditation practices, and homework practices. Girls who do not have their own smartphone will borrow one from Rotterdam University of Applied Sciences.

In order to teach and stimulate girls to discover new healthy and positive shared alternatives for excessive co-rumination and rumination (getting out of autopilot negative reactivity), to stimulate present-moment awareness and appreciation of pleasant shared experiences and interactions, to encourage girls to share their interpersonal responses to pleasant experiences and positive affect with each other, to teach girls to work skillfully with difficult emotions during activities and interactions and to support friendship dyads to incorporate these healthy alternatives in their daily lives, dyadic-friendship girls will participate and will be supported together by the training program and encouraged to use the App yourself happy module “Healthy, joyful dyadic activities” on a daily basis. This module contains 150 healthy and joyful behavioral activation activities for dyadic use with the following categories: Beauty, On the road, Creative, Educative, Game time, Indoor, Outdoor, Mindfulness, Sport, Relax, and Kitchen. Each activity is presented on a photocard with fun and inspiring tips about the preparation of the activity and information about the costs and other important aspects (e.g., “for this activity you need a towel”). Girls will be able to select five activities and share these with their dyad friend via a WhatsApp message.

Friendship dyads will be encouraged in the weekly training sessions to do at least one or two activities each week. These behavioral activities (including the preparations and the reflections on the activities through journaling and reflections during the weekly training sessions) will function as a natural backbone to train several mindfulness skills: being in the present moment (present-moment awareness), conducting random acts of kindness, experiencing joy and gratitude using the ten fingers gratitude practice, using the pleasant experiences calendar and creating more nurturing experiences, and using the unpleasant experiences calendar and diminishing the impact of depleting experiences. In every training session, a new mindfulness practice will be introduced by connecting practice with upcoming activities and skills practicing. The use of these practices will be stimulated by several animation videos with explanations about co-rumination, brain functioning on automatic pilot, and a guided mediation practice. Working with mindfulness skills and practices during the activities and within daily live interactions will be evaluated and reflected on during the online training sessions, and girls will be encouraged and trained to incorporate these mindfulness practices within other and new activities in their daily interactions.

This continuous process of training, reflection, and integration in daily live will be supported by using the journaling module of the app, with daily journaling guided by randomly selected positively formulated mindfulness-based questions (morning, afternoon, and evening), aimed at practicing gratitude and optimism related to (a) daily experiences (always) and (b) positive anticipation on upcoming shared activities and (c) recalling positive memories after completing an activity (during activity planning and after completing activities). This module encourages girls three times a day to actively report on the positive aspects of preparing the activities together with their dyad friend, to actively recall positive memories about the activity as well as aspects of the friendship/sharing and the mindfulness practices after completing an activity. They will be encouraged to share their positive thoughts and positive emotions within their daily journaling and daily interactions and conversations.

During the training sessions, girls will be invited to share their experiences about their cultivated adaptive awareness of together dwelling on negative emotions and the impact of their training practices on awareness of sharing and responding on positive affect. This process will be specifically supported by using the mood tracker and calendar, facilitating girls to reflect on practicing mindfulness-based activities and their daily emotions.

Daily state mood monitoring possibilities (three times a day: morning, afternoon, and evening) are Positive Affect (PA): Cheerful (high arousal), Content (low arousal), Happy (high arousal), Energetic (high arousal), Relaxed (low arousal), and Joyful (high arousal) and Negative Affect (NA): Worried (high arousal), Anxious (high arousal), Low/Depressed (low arousal), Insecure (low arousal), Irritated (high arousal), and Guilty (low arousal). Girls will be asked to report one PA and/or one NA on a 5-point scale, ranging from 0 (*not at all*) to 4 (*very much*). The PA and NA items were derived from work of Barrantes-Vidal and colleagues [62]. A monthly overview of the experienced moods and their intensity will be available within the Mood calendar. Girls will receive daily empathetically formulated prompts on pre-programmed moments (indicated by the girls themselves) for using the mood monitoring, journalling, and diary function of the app and daily rewards (i.e., psychoeducation), offered at completing daily mood monitoring and journaling. This is embedded to boost engagement with the app on a daily basis. It is hypothesized that actively practicing these skills and reflecting on the impact on their emotions and thoughts will explicitly target repetitive negative, judgmental emotions and thoughts in the interactions and communication of the participating girls. Girls will spend a total of no more than 15 min a day completing the mood tracker and diary.

### Care-as-usual (CAU)

In the control condition, girls receive unchanged CAU including teaching practices they are already exposed to in their current schools. No other classroom-based mindfulness interventions will be implemented during the intervention period of the cRCT.

### Train the trainers to deliver the program

The training sessions will be delivered in academic year 2023/2024 (cohort 1) and 2024/2025 (cohort 2) by mindfulness-trained staff members (i.e., PI, PhD students and external staff members). All HFPM trainers have followed an 8-week Mindfulness for Life course at the Oxford Mindfulness Foundation in academic year 2021/2022 or 2022/2023 (seven 2-h sessions per week and one whole day session, supported by a course booklet and several digital guided mindfulness practices to facilitate mindfulness practice during and after the 8-week course). Furthermore, they will all have an established mindfulness practice of more than 6 months before the prevention program starts.

Between October 2023 and January 2024, all trainers will attend a 4-day training program to learn how to deliver the program to the friendship dyads. To maximize fidelity, all trainers have to reach adequate adherence and competence standards before they start teaching trial dyads. This will be conducted through roleplay. During program implementation, all trainers will receive weekly supervision on competence and adherence by the PI Dr. Patricia Vuijk, who also finalized the Mindfulness Frame by Frame course (Oxford Mindfulness Foundation) and the Teacher Training course “Dot-b” (MISP). During the cRCT, competence and adherence will be monitored. Following study procedures of the MYRIAD mindfulness trial regarding the use of the MBI: TAC [63, 64], an independent rater (Ron Weerheijm) and PI Dr. Patricia Vuijk will weekly randomly select a subset of 10% of the videotapes for fidelity monitoring using the MBI:TAC instrument.

### Measures

#### Screening measure (girls)

Self-reported co-rumination will be measured with a subset of items from the Co-Rumination Questionnaire-Short [21] which is based on the original 27-item Co-Rumination Questionnaire [9]. The Co-Rumination Questionnaire-Short consists of 17 items and measures the extent to which girls typically co-ruminate with their best or good same-gender friend, using a 5-point Likert scale, ranging from 0 (*not at all true*) to 4 (*really true*). The five items that will be used for this screening are “When we talk about a problem that one of us has, we try to figure out every one of the bad things that might happen because of the problem,” “When we talk about a problem that one of us has, we spend a lot of time trying to figure out parts of the problem we can’t understand,” “When we talk about a problem that one of us has, we talk a lot about how bad the person with the problem feels,” “When we talk about a problem that one of us has, we’ll talk about every part of the problem over and over,” and “When we talk about a problem that one of us has, we talk about all of the reasons why the problem might have happened.” The English five-item version showed very good reliability of change (RCI = 0.89; [65]). We also calculated reliability statistics for girls (age 13 and 14) living in the Netherlands. Cronbach’s alpha showed good reliability, with *α* = 0.85 and 0.74 for 13- and 14-year-old girls, respectively (unpublished data, data available from the authors). These data will be collected via Qualtrics.

### Primary outcome variables

All data will be collected via Qualtrics.

#### T0, T1, T2, T3, T4, and 1-year follow-up measures T5 (girls)

##### Self-reported negative affect: co-rumination

The original measure [9] to assess co-rumination included 27 items that measure the extent to which youth typically co-ruminate with same-sex friends. Nine content areas are covered, including (a) frequently discussing problems, (b) discussing problems rather than doing other activities, (c) friend encouraging discussion of problems, (d) target child encouraging friend to discuss problems, (e) discussing repetitively the same problem, (f) speculating about cause of problems, (g) speculating about consequence of problems, (h) trying to understand parts of problems, and (i) focusing on negative affective feelings. The items focus on assessing a more extreme form of discussing problems beyond mere self-disclosure.

For the present study, 9 items (1 for each of the topic areas) will be used to assess co-rumination at T0, T1, T2, T3, T4, and T5 1-year follow-up (Co-rumination Questionnaire-short; CRQ-short). These items include: “We talk about problems that my friend or I are having almost every time we see each other”, “When we see each other, if one of us has a problem, we will talk about the problem even if we had planned to do something else together,” “When my friend has a problem, I always try really hard to keep my friend talking about it.,” “When I have a problem, my friend always tries to get me to tell every detail about what happened,” “When we talk about a problem that one of us has, we’ll talk about every part of the problem over and over.,” “When we talk about a problem that one of us has, we talk about all of the reasons why the problem might have happened.,” “When we talk about a problem that one of us has, we try to figure out every one of the bad things that might happen because of the problem.,” “When we talk about a problem that one of us has, we spend a lot of time trying to figure out parts of the problem that we can’t understand.,” and “When we talk about a problem that one of us has, we talk a lot about how bad the person with the problem feels.”

Rose [9] reported that her 27-item measure was unifactorial, and a factor analysis of the 9 items used in Hankin and colleagues [21] similarly revealed a single factor. Internal consistency in this sample with 9 items was a 0.89 at Time 1, 0.91 at Time 2, and 0.91 at Time 3. Girls will respond to the items using a 5-point Likert scale ranging from 0 (*not at all true*) to 4 (*really true*) and scores will be the mean rating of the nine items. Rose and colleagues (2007) reported excellent internal consistency, good test–retest reliability, and validity [11].

##### Self-reported (early-onset risk for) depressive symptoms/disorder

Self-reported degree of depressive symptoms will be measured with the Child Depression Inventory (CDI-2; [66]), which includes 28 items. All items offer three graded options from 0 to 2, of which one should be chosen (e.g., 0 = “*I am sad once in a while*,” 1 = *I am sad many times*,” and 2 = “*I am sad all the time*”) with higher scores indicating more depressive symptoms. Total scores could range from 0 to 56, and a score of 12 or above is considered a clinically relevant score [66]. The CDI-2 has good internal consistency, test–retest reliability, and convergent validity [66].

#### T0 and T4 measures (girls)

##### Observed regulation of negative affect: co-rumination

Co-rumination and the topic of problems (interpersonal problems/non-interpersonal problems) will be observed with the Dutch version of the standardized Problem Talk Task (PTT; [22, 67]. Participation of girls will take place online using the protected Teams-environment of Rotterdam University of Applied Sciences. Both girls will receive links to the Teams-observations via email. The observations will be recorded via Teams. The PTT will start with a short warm-up task. Girls will be asked to plan a party together and to discuss the guest lists, the location, the theme of the party, etc. After the warm-up task, dyads will be told that they will have 16 min to discuss problems or worries they want to share and that are allowed to discuss anything about the problems and/or worries. They will be asked to discuss each friend’s problem and will be told that they are allowed to spend as much time as they want to on each person’s problem/worries. They will be told that, if they finish talking about problems, they could talk about something else.

##### Coding process

All video recordings will be stored and coded in Research Cloud. There will be three major aims of coding. The first is to provide information regarding the degree to which the girls participated in problem talk. The second aim is to provide information regarding how girls responded to friends’ statements about problems and/or worries. The third aim is to provide information about the topics on which girls co-ruminate. A team of two coders (both PhD students) will code the data. The coders will first obtain interrater reliability based on 25% of the interactions and then individually code the remaining interactions.

To determine how often girls participate in problem talk, all thought units that the girls produced will be coded as Own-Problem Statements, Friend-Problem Statements, or Non-Problem Statements. To do this, interactions in the PTT will be first transcribed by students of Developmental Psychology of VU Amsterdam and then segmented by the PhD students into thought units, or logical divisions of speech identified based on contextual and syntactic clues, such as pauses, changes in ideas, or changes in who was speaking [68, 69]. Coders will first classify all thought units according to whether they are Own-Problem Statements (i.e., statements about the speaker’s own problems) or not. Next, the thought units will be classified according to whether they are Friend-Problem Statements (i.e., statements about the speaker’s friend’s problems) or not. Each girl will be given a score for the total number of Own-Problem Statements, Friend-Problem Statements, and Non-Problem Statements that they produce.

Adolescents’ responses to friends’ Own-Problem Statements will be coded next. All turns with at least one Own-Problem Statement will be identified. A turn is defined as a stream of uninterrupted speech with one or more thought units. A turn begins when a girl begins to speak. A turn ends when the friend speaks or there is a pause of approximately 15 s. Like prior coding approaches [68], each thought unit produced by the adolescent in the turns directly following turns produced by the friend that included at least one Own-Problem Statement will be coded into one of the 10 response categories. Six of the categories are considered Positive Engaged responses: Support/Agree (e.g., “I think you did the right thing.”), Question (e.g., “When did it happen?”), Related Experience (statement on problem topic about one’s own experience that is related to the statement from the original speaker, e.g., “I feel sad when she doesn’t return my calls too.”), Information/ Opinion (new information about problem, e.g., “And her parents let her stay out past midnight” or an opinion presented in a relatively neutral manner, e.g., “I thought he worked hard on that.”), Acknowledge/Prompt (conveys the listener is paying attention, with or without explicitly encouraging the speaker to continue, e.g., “Uh-huh,” “Oh,” “Keep talking.”), Advice Giving (e.g., “You should call her.”).

Three of the responses are Negative: Changing the Subject (statements that are not focused on the problem topic, e.g., “I’m hungry” or that focus on the adolescent’s experience with the problem topic in a manner that draws attention away from original speaker, e.g., “Well, the person who she ignores the most is me!”), Minimization/Non-Support (explicitly non-supportive statements or statements that convey the problem is less important than the speaker portrayed it to be, e.g., “Everyone hates it when you say that,” “That’s not a problem.”), and Silence/No response (no response and a break in the conversation for about 15 s or more). The final response is Humor (conveys humor, joking, or non-hostile sarcasm). There is an Other category for thought units that were unintelligible or had no substantive meaning (e.g., “Well,” “Um”); the Other category will be not used in analyses.

Summed scores will be computed for each girl that are the total number of responses of each type that the adolescent produce. The decision to use summed scores for the analyses (as opposed to proportion scores, i.e., the percent of responses of each type out of the total number of responses) is based on the idea that the total number of experiences that adolescents have in which they hear positive engaged, negative or humorous response statements (in other words, the cumulative experience of hearing these responses) should have a particular critical influence on friendships.

Finally, all the problem-focused thought units will then be assigned one of the topic codes. The topic codes were generated a priori based on the literature reviewed regarding challenges during adolescence. Three topic codes are interpersonal: family, peers (nonromantic), and romantic interests. Three are not interpersonal: academics, athletics and other extra-curricular activities, and jobs and money. Finally, each problem-focused thought unit will be timed. The number of seconds that friend dyads spent on each problem topic will be summed. For each step, 25% of the dyads will be double coded. Interrater reliability will be computed separately for T0 and T4.

### Secondary outcome variables

#### T0, T1, T2, T3, T4, and 1-year follow-up measures T5 (girls)

##### Self-reported (early-onset risk for) generalized anxiety symptoms/disorder

Self-reported generalized anxiety disorder will be measured with the Dutch version of the subscale generalized anxiety disorder (GAD) of the Revised Children’s Anxiety and Depression Scale (RCADS; [70]). This scale has six items (e.g., “I worry about things”) and assesses generalized anxiety on a 4-point scale (0 = *never*, 3 = *always*). The RCADS has good psychometric properties and has demonstrated excellent reliability and validity [70, 71]. We will use the official Dutch translation of the RCADS, which is freely available. The Dutch version of the RCADS—subscale GAD—showed good internal consistency, validity, and sensitivity to change [72].

##### Self-reported problem anxiety talk

Self-reported problem anxiety talk will be measured with the Dutch version of the Problem Anxiety Talk Scale (PATS; [73]). The items are developed to reflect (1) repetitive focus on anxiety‐related topics; (2) threatening interpretations of ambiguous situations; (3) anticipating the likelihood of future negative stressful events; and (4) lack of confidence about successfully coping with stressors. Instructions prompt girls to report on specific verbal interchanges during conversations with their same‐gender best friend with the phrase “When we talk about our worries…” Girls will rate the extent that each item reflects their conversations on a 5‐point Likert scale ranging from 1 (*not at all true*) to 5 (*very true*). Examples of items include “Once I start discussing my worries with my friend, we find it hard to stop,” (i.e., repetitive focus on anxiety‐provoking topics), “My friend often helps me think of possible risks or bad things that might happen,” (i.e., threatening appraisal), “When we discuss bad things that could happen, we talk as if the bad thing will definitely happen” (i.e., likelihood of negative events), and “When my friend and I discuss my worries I start to question my ability to handle future problems. (i.e., lack of confidence in coping skills). The PATS is scored as the sum of all scale items, ranging from 12 to 60 [73]. The PATS demonstrates strong internal consistency (Cronbach’s *α* = 0.89).

##### Self-reported positive and negative affect

Self-reported positive and negative affect will be measured with the Dutch version of the Positive and Negative Affect Schedule for Children (PANAS-C; [74, 75]). The PANAS-C consists of the two subscales Positive Affect (15 items, e.g., energetic) and Negative Affect (15 items, e.g., nervous). Participants are instructed to indicate for each item how often they have experienced that feeling over the past few weeks. Items are scored on a scale ranging from 1 (*very slightly or not at all*) to 5 (*extremely*). The internal reliability of this Dutch version was moderate to good for the Positive Affect subscale (Cronbach’s *α* between 0.66 and 0.83) and for the Negative Affect subscale between 0.67 and 0.81. We will create a dummy variable that represents the type of day (i.e., 0 = *weekend day*, 1 = *weekday*). For the time of day, we will create different dummy variables that represent whether the assessment occurred during the morning (i.e., *morning* = 1, *afternoon and evening* = 0), afternoon (*afternoon* = 1, *morning and evening* = 0), or evening (*evening* = 1, *morning and afternoon* = 1).

##### Self-reported interpersonal responses to positive affect

Self-reported interpersonal responses to positive affect will be measured with the Co-Dampening and Co-Enhancing Questionnaire (CoDEQ; [76]). Items were constructed to assess interpersonal dampening and enhancing responses to happy feelings within dyads and consist of 18 items; nine items will measure co-enhancing and nine items will measure co-dampening. For intrapersonal dampening, the following responses to positive affect are described: thinking about the fleetingness of positivity, thinking about worries, focusing on negative aspects of the positive affect or event, making upward social comparisons (i.e., how others are even better off than you), making external attributions (e.g., thinking “it was just luck”), and starting to think about past negative events. Co-enhancing items were based on the following enhancing responses: behavioral display, focusing on positive feelings (e.g., thinking about how energetic one feels), thinking about positive past and future events, making downward social comparisons (i.e., comparing yourselves to those who are less fortunate), and thinking about positive self-qualities such as the ability to achieve whatever you desire. Respondents must indicate how often they respond in the described way when one of them feels glad or happy and they are talking about this. The rating scale has four response options: *almost never* (1), *sometimes* (2), *often* (3), and *almost always* (4). Cronbach’s alphas were 0.84 and 0.86 for co-enhancing and co-dampening respectively [77].

##### Self-reported quality of the friendship with the dyad friend and investments in the interpersonal relation

Self-reported support quality of the friendship with the dyad friend and investments in the interpersonal relation will be measured with the Dutch version of the Network of Relationships Inventory (NRI; [78]), which includes 36 items. The NRI has seven subscales. The subscale Support consists of eight items. Adolescents and their best friend were instructed to take each other in mind while answering items such as: “How much does your best friend really care about you?”. The subscale Relative Power consists of six items (“To what extent is your friend the boss in your relationship?”). The subscale Negative Interaction consists of sex items (“Are you and your friend annoyed by each other’s behavior?”). The subscale Seeks Safe Haven consists of three items (“To what extent do you visit your friend when you are upset?”). The subscale Provides Safe Haven consists of three items (“To what extent does your friend visit you when she is worried about something?”). The subscale Seeks Secure Base consists of three items (“To what extent does your friend support you in the things you do?”). Finally, the subscale Provides Secure Base consists of three items (“To what extent do you support your friend in the things she does?”). Responses will be rated on a 5-point Likert scale 0 (*little or none*) to 4 (*the most*). The English version of the NRI shows good psychometric properties in adolescent samples, such as high internal consistency and moderately high stability over a 1-year period [79]. Furthermore, in a Dutch sample that used a shorter version of the NRI friend-scales, it was shown that the internal consistencies were high for all variables (Cronbach’s alpha range *α* = 0.82–93) and that the factor and construct validity of the NRI are adequate [80].

##### Self-reported interpersonal reactivity to dyad friend personal distress

Self-reported interpersonal reactivity to dyad friend personal distress will be measured with the Dutch version of Interpersonal Reactivity Index for Personal Distress (IRI-PD; [81, 82]). The IRI-PD consists of seven items (e.g., I’m usually effective in managing stressful situations) and the rating scale ranges from (0) = *describes me not at all* to (4) *describes me very well*. Cronbach’s alpha of observed mean scores showed acceptable values, ranging from 0.67 to 0.87. The psychometric qualities of the Dutch version of the IRI-PD questionnaire were examined by De Corte and colleagues [82]. Cronbach’s alpha was 0.77 [82].

##### Self-reported anhedonic symptoms

Self-reported anhedonic symptoms will be measured with the Leuven Anhedonia Self-report Scale (LASS; [83]). The items tap the consummatory (i.e., reduced pleasure in ongoing experiences), anticipatory (i.e., the diminished pleasure from anticipation to a future positive event), and the motivational (i.e., the decreased drive or motivation to pursue positive outcomes or reward) aspects of anhedonia. Participants will be asked to rate twelve statements according to the last 2 weeks. Example items are: “I found little pleasure in things that I used to enjoy,” “I could get really excited in advance about fun things,” and “I was motivated to do all kinds of things.” The rating scale ranges from *completely untrue* (1) to *completely true* (5). Internal consistency of the total scale is good (*α* = 0.81; [76]).

##### Self-reported mastery

Self-reported mastery will be measured with the Dutch version of the Pearling Mastery Scale (PMS; [84]). The PMS questionnaire contains seven items, each of which is scored on a 4-point Likert scale (1 = totally disagree, 4 = completely agree). A high score reflects a higher degree of mastery. The reliability is adequate (Cronbach’s *α* = 0.78; [85]).

#### T0 and T4 measures (parents)

##### Parental health care use of their child/family

Parental health care use of their child/family will be measured by three questions about whether the child and/or family received health care for psychosocial, emotional, or behavioral problems of the child in the last 12 months, the underlying cause(s), and the duration of the care.

### Moderator

#### T0, T1, T2, T3, T4, and 1-year follow-up measures (girls)

##### Self-reported self-control

Self-reported self-control will be assessed using the Dutch version of the 5-item Self-Control Measure (SCM; [86, 87]). Items will be rated on a 5-point Likert scale (“*not true at all*” = 1 to “*completely true*” = 5). Example items are “I have trouble saying no” and “I do certain things that are bad for me, if they are fun.” A higher sum score will reflect higher self-control (Cronbach’s *α* = 0.62 [88]).

### Mediators

#### T0, T1, T2, T3, T4, and 1-year follow-up measures (girls)

##### Self-reported trait mindfulness

Self-reported trait mindfulness will be measured with the Dutch adolescent version of the Comprehensive Inventory of Mindfulness Experiences (CHIME-A; [89, 90]). The CHIME-A assesses eight different aspects of mindfulness: awareness of internal experiences, awareness of external experiences, acting with awareness, accepting and nonjudgmental attitude, non-reactive decentering, openness to experiences, awareness of thought’s relativity, and insightful understanding. Its 24 items are scored on a 6-point Likert scale with higher scores indicating greater mindfulness skills. The subscales of the Dutch version of the CHIME-A have acceptable psychometric properties with Cronbach’s alphas ranging from 0.67 to 0.77 [90].

##### Self-reported emotion regulation

Self-reported emotional regulation will be measured with the Dutch version of the Difficulties in Emotion Regulation Scale (DERS; [91, 92]). The DERS is a 36-item self-report questionnaire developed to assess individuals’ identification, understanding, and modulation of their own emotions (Gratz & Roemer, 2004). Items are scored on six scales, labeled Lack of Emotional Awareness (6 items), Lack of Emotional Clarity (5 items), Difficulties Controlling Impulsive Behaviors When Distressed (6 items), Difficulties Engaging in Goal-Directed Behavior When Distressed (5 items), Nonacceptance of Negative Emotional Responses (6 items), and Limited Access to Effective ER Strategies (8 items). Items are scored on a 5-point scale ranging from 1 (*almost never*) to 5 (*almost always*). Subscale scores will be obtained by summing up the corresponding items. The subscales of the Dutch version of the DERS have satisfactory to high psychometric properties with Cronbach’s alphas ranging from 0.72 to 0.87. [92].

### Implementation measures

#### T1, T2, T3, T4

In order to study how implementation impacts the hypothesized effects of the HFPM program, the following variables will be studied:

1. *Program dosage*, i.e., how much of the program has been followed, will be measured by the total amount of complete delivered training sessions within each dyad (one question) and the percentage of dyadic attendance to the sessions (one question). Data on both variables will be collected by the trainers, who will have to fill out a digital form after each training session.
2. *Participant responsiveness*, i.e., the degree to which the program engages and stimulates the interest of the participants, will be measured by seven questions assessing rates of usefulness, perceived benefits, engagement, enjoyment, helpfulness, intentions to apply to daily life, and perceived success in applying to daily life. Girls will be asked to rate their answers on a 5-point Likert scale, ranging from “*not at all*” to “*very much*.” Data will be collected by digital questionnaires immediately following each training session.
3. *The extent of participants’ self-reported practice outside of the training sessions* will be measured by the percentages of (1) completed activities (i.e., back-end data collected by the app), (2) completed daily state mood monitoring activities (i.e., back-end data collected by the app), and (3) completed daily mindfulness-based questions (i.e., back-end data collected by the app). Moreover, data on self-reported practice outside of the training sessions will be collected by asking girls to fill out digital questionnaires after each training session about the amount of self-reported mindfulness practices within their dyadic conversations, ranging from low [*once a week or less*], medium [*three times a week or less*], and high [*at least three times a week*].
4. *Program fidelity*, i.e., the extent to which the delivered program corresponds to the original program will be collected using self-administered digital forms completed by the trainers after each lesson, reporting whether the scheduled activities were delivered (yes or no) and whether the trainers (yes or no) altered any activities. The fidelity forms will list all activities planned for each training session, based on the training manual. The PI will instruct the trainers on how to complete the form. Each item will have data on the percentage of activity completeness (i.e., the numerator will be the number of activities delivered, and the denominator will be the total number of activities planned), and percentage of alterations (i.e., the numerator will be the activities instructors reported changing, and the denominator will be the number of activities planned). A fidelity variable for each dyad will be calculated as follows: fidelity = % completeness × (1 − % alteration). Dyads will then be divided into two groups according to the level of fidelity: those that received ≥ 80% of the proposed activities will be considered to have completed the program, whereas those that received < 80% of the activities will be considered to have incomplete implementation.
5. *Program quality*, i.e., how well the different program components are delivered, will be observed via the recorded Teams-training sessions (protected Teams-environment of Rotterdam University of Applied Sciences) with a standardized measure, the Dutch version of the Mindfulness Based Interventions – Teacher Assessment Criteria; MBI:TAC; [64, 93]). The six teaching competence domains within the MBI:TAC are (1) coverage, pacing, and organization of the session curriculum; (2) specific interpersonal relational skills; (3) skillful guiding of formal mindfulness meditation practices; (4) specific methodologies for conveying the course themes through interactive inquiry, (5) group dialog and didactic teaching; and (6) effective holding of the group teaching/learning environment. This instrument will also be used in the training program including supervision during which the criteria are used as a developmental tool to offer clear feedback to the trainers and to identify foci for development.
6. *Treatment contamination*, i.e., monitoring of the control group, will be measured by an online questionnaire for parents at T0, T2, and T4 with one question: “Did your child attend a mindfulness-based course in the last two or three months, and if yes, please mention the name of the course”.

### Descriptives

#### T0 measure (parents/main caretakers)

##### Socioeconomic status

Following recent studies in the Netherlands [94, 95], parental education level will be used as an indicator of socioeconomic status (SES), which has been regarded the most powerful indicator of SES [96–98]. To this end, the primary caregiver will report the education level of the mother and the father of each participant. Educational levels will be rated according to the Dutch Standard Education Classifications (Statistics Netherlands, 2021), which corresponds to the International Standard Classification of Education (ISCED; [99]). Following the ISCED classifications, parental education levels will be coded using an 8-point scale, with education levels including the following: 0 = *no education/early education*, 1 = *primary education*, 2 = *lower secondary education* (e.g., junior secondary school, middle school, junior high school), 3 = *upper secondary education* (e.g., senior secondary school, [senior] high school), 4 = *post-secondary non-tertiary education* (e.g., technician diploma, primary professional education), 5 = *short-cycle tertiary education* (e.g., [higher] technical education, higher/advanced vocational training, associate degree), 6 = *bachelor’s degree or equivalent*, and 7 = *master’s degree, equivalent or higher*. Parental education level will be based on the highest completed parental education level per household. That is, if a child has one parent with upper secondary education (i.e., 3) and another parent with a bachelor’s degree (i.e., 6), then we will code this child’s parental education with bachelor’s degree (i.e., 6).

##### Ethnicity

Ethnicity will be dichotomized as Dutch or non-Dutch based on where parents/main caretakers and their child is born.

*Intervention status* will be dummy-coded (0 = *control*, 1 = *HFPM program*).

## Study procedures

### Measures

#### Questionnaires T0, T1, T2, T3, T4, and 1-year follow-up T5

Girls, parents/main caretakers, teacher-mentors, and trainers will receive the online questionnaires hosted by Qualtrics at T0, T1, T2, T3, and T4 and at 1-year follow-up T5 via email by well-trained PhD students. After 1 week, participants will be receiving a reminder via email, followed by contact by telephone after another 3 days. Reminders will be sent by the teacher-mentors and PhD students will be responsible for the telephone calls. Table S1 describes the burden (in minutes) for girls in both groups, Table S2 for parents/main caretakers, and Table S3 for trainers.

#### Observations co-rumination T0 and T4

For a detailed description of the PTT, please see the section “Primary outcome variables.” The duration of the PTT is 30 min. The video recordings will be conducted by trained research assistants through Teams (students Developmental Psychopathology of the Vrije Universiteit Amsterdam) and will be supervised by both PhD students.

### Incentives

#### Screening

Girls who participate in the screening will receive a present worth 2.50 Euro.

#### T0 t/m T5 measures

Girls will receive a voucher of 10.00 Euro after completing each measurement.

#### Participation in the prevention program

To encourage dyadic app use during the period of November 2023 and April 2025, friendship dyads will build up a credit of 2.50 euros per week when they both complete daily monitoring (three times a day) and journaling (three times a day) for six out of the seven weekdays. Study personnel will email parents and girls weekly regarding the number of times girls used the app that week, and whether they will earn the weekly credit.

### Trial status

Protocol version 2, dd. 10–11-2023. Recruitment of schools started in December 2022, with recruitment of girls beginning in August 2023. Recruitment of girls will be finalized December 2025.

## Data analysis plan

Note that only the principal features of the statistical methods for primary and secondary outcomes are reported in this section. For an elaborate description of the analyses methods used to test the hypotheses, please see the Statistical Analyses Plan (SAP) in Supplementary Materials.

### Statistical methods for primary and secondary outcomes

Hypothesis 1 “Girls in the intervention group will have a greater reduction in co-rumination about distress and difficult emotions and feelings, (and thereby) internalizing symptoms and negative affect during the intervention period, immediately after the intervention period and after 1-year follow-up, relative to girls in the control condition” will be tested via (a) a multi-level parallel process, dual latent growth curve model (with co-rumination measured via self-report) and (b) a multi-level autoregressive cross-lagged model (with co-rumination measured via observation [100, 101]).

For analysis (a) with co-rumination measured via self-report (T0 to 1-year follow-up T5), we will use multi-level parallel process, dual latent growth curve (LGM) models, with intervention arm as the exogenous predictor variable, growth rate of co-rumination as the mediating variable, and growth rate of internalizing symptoms and negative affect as (correlated) outcome variables. See Fig. S3 for a simplified visual representation of this model.

For analysis (b) with co-rumination measured via observations (T0 and T4), we will use a multi-level autoregressive crossed-lagged model, with intervention arm as the exogenous predictor variable (relative change from T0 to T4 in) co-rumination as the mediating variable and (relative change from T0 to T4 in) internalizing symptoms and negative affect as (correlated) outcome variables. See Fig. S4 for a simplified visual representation of this model.

Hypothesis 2 “Girls in the intervention group will have (a) a later onset of depressive symptoms or a later onset of depressive disorder and (b) less dyadic depression contagion during the intervention period, immediately after the intervention period and after 1-year follow-up, relative to girls in the control condition” will be tested using discrete-time survival analysis [102] and via actor-partner interdependence modeling [103], respectively.

Specifically, discrete-time survival analysis (DTSA) will be used to investigate whether girls in the intervention group have a later onset of depression symptoms or disorder compared to girls in the control arm. Note that this hypothesis focuses on individual rather than dyadic outcomes. To adjust for individuals clustered within dyads, we will adjust the standard errors using a sandwich estimator [104]. Furthermore, a prospective change, Actor-Partner Interdependence Model (APIM) will be used to investigate whether dyads in the intervention condition will experience less dyadic depression contagion compared to dyads in the control arm from T0 to 1-year after follow-up. We will use multi-group comparisons to test whether contagion effects are less strong in the intervention arm compared to the control arm.

Hypothesis 3 “Girls in the intervention group will demonstrate (a) less anxiety problem talk and will have a (b) later onset of anxiety symptoms or a later onset of anxiety disorder and (c) less dyadic anxiety contagion during the intervention period, immediately after the intervention period, and after 1-year follow-up, relative to girls in the control condition” will be tested using multi-level LGM, DTSAs, and multi-group APIM, respectively.

Specifically, a multi-level LGM with intervention arm as the exogenous predictor variable and growth rate of anxiety problem talk as the outcome variable will be used to test whether girls in the intervention arm demonstrate less anxiety problem talk compared to girls in the control arm. Furthermore, DTSAs of anxiety symptom onset and anxiety disorder onset with intervention condition as an exogenous predictor will be used to test whether girls in the intervention group have a later onset of anxiety symptoms or disorder compared to girls in the control arm. Lastly, a multi-group (i.e., intervention versus control condition) APIM will be used to investigate whether dyads in the intervention condition will experience less anxiety contagion compared to dyads in the control arm from T0 to 1-year after follow-up.

Hypothesis 4 “Girls in the intervention group will experience better friendship quality, higher levels of positive affect and higher levels of interpersonal responses to positive affect of the dyad friend, during the intervention period, immediately after the intervention period and after 1-year follow-up, relative to girls in the control condition” will be tested using multi-level LGMs for each outcome, respectively. Specifically, three multi-level LGMs with intervention arm as the exogenous predictor variable and growth rate of friendship quality, positive affect, and interpersonal responses to positive affect as the (potentially correlated) outcome variables, will be used to test whether girls in the intervention arm experience more improvements in friendship quality, a faster growth in positive affect and faster growth levels of interpersonal responses to positive affect, compared to girls in the control arm.

Hypothesis 5 “The hypothesized intervention effects on co-rumination will be mediated by the development of mindfulness skills, emotion regulation skills, and problem-solving skills during the intervention period, immediately after the intervention period and after 1-year follow-up” will be tested using (a) a multi-level parallel process LGMs (for self-reported co-rumination) and (b) a multi-level autoregressive cross-lagged model (with co-rumination measured via observation). Specifically, for the model where co-rumination is measured via self-report, a multi-level (LGM) model, with intervention arm as the exogenous predictor variable, growth rate of mindfulness skills, emotion regulation skills, and problem-solving skills as the mediating variables and growth rate of co-rumination as the outcome variable. Furthermore, for analysis of the model with co-rumination measured via observations (T0 and T4), we will use a multi-level autoregressive crossed-lagged model, with intervention arm as the exogenous predictor variable, (relative change from T0 to T4 in) mindfulness skills, emotion regulation skills, and problem-solving skills as the (correlated) mediating variables, and (relative change from T0 to T4 in) co-rumination as the outcome variable.

Hypothesis 6 “The hypothesized intervention effects on co-rumination will be moderated by self-control: girls with more developed self-control skills will demonstrate greater intervention effects immediately after the intervention period and after 1-year follow-up, relative to girls in the control condition” will be tested (a) a multi-level parallel process moderation LGM (for self-reported co-rumination) and (b) a multi-level autoregressive cross-lagged model (with co-rumination measured via observation).

Specifically, the hypothesis that the intervention effects on self-reported co-rumination development will be moderated by development in self-control will be tested using a multi-level LGM. In this model, intervention arm is an exogenous predictor of the (between-level) growth curve of outcome variable co-rumination and growth in self-report from T0 to 1-year after follow-up is the moderator. Thus, interactions between the slope of the moderator self-control and the slope of the outcome variable co-rumination will be added to the main effect model to test whether the path between intervention condition (intervention versus control group) and self-reported co-rumination development is moderated by growth in self-control [105]. Furthermore, for the analysis with co-rumination measured via observations (T0 and T4), we will use a multi-level autoregressive crossed-lagged model, with intervention arm as the exogenous predictor variable, (relative change from T0 to T4 in) self-control as the moderating variable, and (relative change from T0 to T4 in) co-rumination as the outcome variables.

Hypothesis 7 “Girls in the intervention group will experience less anhedonic symptoms, will experience greater feelings of mastery and will show less health care use, immediately after the intervention period and after 1-year follow-up, relative to girls in the control condition” will be tested using multi-level LGMs for each outcome, respectively. Five multi-level LGMs with intervention arm as the exogenous predictor variable and growth rate of anhedonic symptoms, feelings of mastery, and health care use as the (potentially correlated) outcome variables, will be used to test whether girls in the intervention arm experience less anhedonic symptoms, greater feelings of mastery, and less health care use over time, compared to girls in the control arm.

Hypothesis 8 “Girls in the intervention group will demonstrate a change in subjects discussed: girls will demonstrate less problem talk about interpersonal problems and shorter periods of interpersonal problem talk immediately after the intervention period and after 1-year follow-up, relative to girls in the control condition” will be tested using Latent Transition Analysis (LTA). Via LTA it can be tested whether girls transition from one class (e.g., a class consisting of girls who engage a lot in interpersonal problem talk; class 1) to another class (e.g., a class consisting of girls who do not engage a lot in interpersonal problem talk; class 2) over time (i.e., between T0 and T4). Via multi-group testing we will investigate whether the probability of changing from class 1 to class 2 is higher for girls in the intervention arm compared to girls in the control arm.

Hypothesis 9 “The hypothesized intervention effects on (the onset) of depression and anxiety symptoms or disorders and depression contagion and anxiety contagion are differently related to changes in interpersonal components of the conversations between girls” will be tested via a joint LTA and DTSA model. First, classes of girls who do and who do not change in their amount and period of interpersonal subjects discussed will be identified via an LTA. Next, it will be investigated whether the resulting classes retrieved from the LTA (i.e., probability of class-membership for the identified transition classes) is a mediator between the intervention effect and the DTSA model of the onset of depression and anxiety symptoms or disorders.

All analyses will be conducted in structural equation modeling program Mplus (v 8.7 or higher; [106]). Model fit will be determined using the comparative fit index (CFI; critical value ≥ 0.95), the root mean square error of approximation (RMSEA; critical value ≤ 0.06), and the standardized root mean square residual (SRMR; critical value < . 08; [107]). When appropriate, standard errors will be adjusted for clustering of dyads within schools using a sandwich estimator [104]. For hypotheses that include mediation analysis (hypothesis 1 and 5), we will use 10,000 bootstrap resamples with replacement and bias-corrected 95% confidence intervals to test these indirect effects [108]. When appropriate, differences in (indirect) pathways between the intervention and the control arm will be estimated using the DIFFtest option in Mplus (when using the weighted least squares means and variance adjusted (WLSMV) estimator) or the Satorra Bentler chi-square difference test [109]; when using the maximum likelihood estimator with robust standard errors (MLR) estimator).

### Handling of protocol non-adherence and missing data

The way the questionnaires are administered online makes it impossible to skip a question. Participants who do not complete a questionnaire will receive one or more reminders. Missing data during observations is also not possible. Missing data during the follow-up period (e.g., due to unavailability during a certain measurement wave or due to dropout) will be handled using Full Information Maximum Likelihood (FIML) estimation.

### Interim analyses

No interim analysis or stopping guidelines will be applied.

## Ethics

### Regulation statement

The study will be conducted according to the principles of the Declaration of Helsinki, current version 2008, and is in accordance with the Medical Research Involving Human Subjects Act (WMO, Sect. 4 minors), guidelines formulated in “Toetsing van onderzoek met minderjarige proefpersonen” (CCMO, 2017) and the Code of Conduct relating to expressions of objection by minors participating in medical research [110].

### Objection by minors or incapacitated subjects

The study is aimed at female adolescents of 13, 14, and 15 years of age. Parents/main caretakers have to sign a written consent, but only if their child is willing to participate. Adolescents have to sign an informed consent, in addition to their parents/main caretakers. Signed informed consent from both parents/main caretakers is crucial for participation. Participants are free to withdraw from the study at any time, without further consequences. There will be specific attention for resistance or objection to perform the assessments with minors. In case of resistance, study participation will be terminated. Examples are (1) the adolescent or/and her best friend look upset, (2) the adolescent or/and best friend end up arguing, and (3) the adolescent or/and best friend indicate that we need to stop video recording or want to end study participation. Researchers will adhere to the Code of Conduct for resistance in minors [110].

### Benefits and risks assessment, group relatedness

The burden and risks for girls associated with participation in the study are seen as minimal given that the study does in no way interfere with regular education of the girls and is focused on natural occurring interactions and activities within girls’ close friendships. The current study offers girls the opportunity to join a study focused on gaining more knowledge about the prevention of excessive co-rumination processes and internalizing problems in adolescent girls.

### Withdrawal of individual subjects

Parents/main caretakers, the girls and their best friend can withdraw from the study at any time for any reason if they wish to do so without any consequences. This is also explicitly formulated in the participant information sheet for parents/main caretakers and adolescents. It will be stressed that withdrawal from the study does not have any consequences for how they are being treated at school or elsewhere. In the case that parents/main caretakers and adolescents decide to leave the study, this means that no further information will be gathered from the adolescent and their friend from that point onwards. Data collected in previous waves will be removed upon the adolescent’ or parents’ explicit request. It is expected that withdrawal is minimal in this study because of minimal burden put onto the girls. Through good coaching by the PI and fulltime availability of the researchers, withdrawal will be minimized where possible. Dropouts will be replaced with a maximum of 16 dyads (*n* = 32), and only between T0 and the end of Phase 1 (see Fig. 1) of the prevention program. No follow-up will be conducted with subjects withdrawn from the study.

### Premature termination of the study

#### Early termination of the study

Previous studies with the PTT [22, 34] indicated that there are minimal risks of participating in the PTT. Furthermore, previous mindfulness-based studies showed minimal risks of participating in app-based mindfulness training for study purposes [111]. To be able to work skillfully with girls/dyads experiencing expected and unexpected unpleasant experiences and potential harm, we will follow the framework recommendations formulated by Baer and colleagues [112], which will be described in more detail in the following section. Therefore, no criteria are formulated for premature termination of the study.

#### Negative and serious adverse events

##### Participation in the PTT

In advance of the PTT, parents/main caretakers will be asked to stay at home during the online measurements to assist their daughter and provide support if needed. During the PTT, the trained researchers and research assistants will adhere to the Code of conduct for resistance in minors and the Observation protocol. Girls will also be told that they are free to withdraw from the task at any time, without further consequences. They will also be told that all information they share during the PTT will be treated confidentially, but that the researchers should terminate the task when there are doubts about the safety of the participants and that, in that case, parents/main caretakers or school professionals will be informed (see below for details). Girls will be informed that this will be the case when they talk about suicide, self-injury, sexual or physical abuse, substance abuse, or eating disorders during the task. Girls will be explained that this rule will prevent them from getting upset during the task. Girls will have to provide written informed consent for this part of the task separately before the task will start. In case of refusal, the PTT will not take place.

The researcher will stop the PTT immediately when the dyad starts discussing these problems and/or when the girls are clearly distressed or upset. The researcher will explain that the safety of the participants is most important and that the goal of the task is not to upset them or to discuss severe problems. The researcher will intervene by asking the girls how they are feeling, comforting them, and by offering them to take a break. If necessary, the girls can also seek comfort from their parents/main caretakers. Depending on how the girls feel after the break, the task will either be continued or terminated. This will be registered in the researcher’s logbook which is accessible across the team.

When severe problems are discussed, or when the girls got upset, the coordinating researcher will immediately (by no contact within 24 h) contact the PI. The PI will contact the independent health care professional of Youz Rijnmond in case of severe problems. Depending on the situation, parents/main caretakers of both participants of the dyad or the teacher-mentor will be informed by the PI, depending on the specific topic the dyad discussed. The Principal Investigator will inform parents/main caretakers in situations of non-family-related acute problems and will advise them to contact the family doctor or the independent health care professional. In case of severe family-related problems, the PI will call inform the teacher-mentor. We will inform the mentor in case of family-related acute problems, so that the school will be able to decide to use the Veilig Thuis Protocol. In severe cases, the Independent Health Care Professional will also be consulted by the PI.

In all situations, we protect the privacy of all participants. This implies that we only should inform parents/main caretakers or the mentor more in depth when the participant initiated the conversation about suicide, self-injury, sexual or physical abuse, or eating disorders. In these cases, the dyad will be excluded from the study. In case of resistance during the task, study participation will also be terminated. This will be the case when (1) one of the participants behaves very upset; (2) the dyad will end up arguing; or (3) one of the participants within a dyad indicate that we need to stop video recording. The dyad will be excluded from the study and parents/main caretakers will be informed by telephone. After finishing the task, participants will be told that they can contact the independent health care professional when they feel upset. We will provide the telephone number of the independent healthcare professional.

##### Participation in the CDI-measures at T0 to T5

Parents/main caretakers will be informed by the PI when girls score > 12 on the CDI-2 (cut-off score for depression) by telephone and will be advised to contact their family doctor or the independent healthcare professional for advice.

##### Participation in the RCADS-measures at T0 to T5

Parents/main caretakers will be informed by the PI when girls will have a T-score > 65 on the RCDAS (cut-off score for anxiety disorder) by telephone and will be advised to contact their family doctor or the independent healthcare professional for advice.

##### Participation in the intervention condition (participating in the online training sessions and using the app)

Participating in the online training sessions

In this study, the potential for harm is defined as sustained deterioration in a girl’s functioning that is *attributable to the prevention program* [113]. To be able to manage (potential) harm caused by the online training sessions skillfully and in a protocolled manner, a risk management protocol has been developed by the PI and research team to provide a consistent approach to the identification, reporting, and follow-up of risks related to participation in the intervention condition. Following study procedures of Baer and colleagues [112], several questions will follow each training session and relating to unpleasant experiences during the sessions, perceived harm from the sessions, and support for any difficult experiences. Some will use Likert scales whereas others will request free-text responses. A written introduction to these questions noted that the practice of mindfulness can increase awareness of the full range of human experiences, including difficult thoughts, emotions, and sensations. Girls then will be asked how often they had such experiences during the course (with response options ranging from “*never*” to “*daily or almost daily*”) and how upsetting these experiences were (response options ranging from “*not at all*” to “*extremely*”). An open text response question will ask the girls to describe their unpleasant experiences during the mindfulness training in more detail. Next, harm will be defined for girls as being “worse off, in any way, after the course, then you would have been if you hadn’t done the course.” Girls will be asked how harmful the course is for them (response options ranging from “*not at all*” to “*extremely*”) and a free-response question will ask them to describe the harm in more detail. Finally, they will be asked if they had sought support for any difficult experiences and, if so, from whom and how helpful the support was. After each training sessions, trainers will send the questionnaires to the PI and she will contact girls who reported difficulties during the sessions within max. 3 days after the session, and she will provide appropriate support, eventually after consulting the independent health care professional.

It is also possible that there will be deteriorations in girls’ functioning as a result of *frictions with the dyad friend* during the intervention period. The training program consists of fourteen online meetings in which the dyads participate together. Each dyad has its own trainer who provides training during all meetings. Every meeting the trainers discuss with the dyads how the cooperation with the app and the homework assignments went in the previous practice period. These discussions have a signaling function: trainers will invite the girls to share all their experiences. We assume that girls will share any problems regarding, for example, their motivation and any frictions or disagreements with the trainers (or that these are observable by the trainers based on verbal and non-verbal behavior during the meetings), so that solutions can be found together. If necessary, trainers may also seek general advice with the teacher-mentors. These possible problems, solutions, and results are shared by the trainers during the intervisions, so that trainers can offer uniform advice as much as possible.

The use of the app is monitored at the individual level by the researchers. When girls do not fill in the diary and moodtracker for more than 3 days in a row, the researchers will pass this on to the trainers, because this may indicate motivation problems, whether this is caused by mutual friction or something else. But girls may also be sick, for example, which causes them to lose track of the app for a while. During the next meeting, the trainers will discuss the causes of non-response with the dyads and jointly look for solutions. Together with possible solutions and results, this information will also be shared by the trainers during the intervisions.

When girls talk about suicide, self-injury, sexual or physical abuse, substance abuse, or eating disorders during the online training sessions, the trainer will stop the session. In these cases, the coordinating researcher will immediately (by no contact within 24 h) contact the PI. The PI will contact the independent health care professional in case of severe problems. Depending on the situation, parents/main caretakers of both participants of the dyad or the school-mentor will be informed by the PI, depending on the specific topic. The PI will inform parents/main caretakers in situations of non-family-related acute problems and will advise them to contact the family doctor or the independent health care professional. In case of severe family-related problems, the PI will call inform the school-mentor. We will inform the mentor in case of family-related acute problems, so that the school will be able to decide to use the Veilig Thuis Protocol. In severe cases, the independent health care professional will also be consulted by the PI. In all situations, we protect the privacy of all participants. This implies that we only should inform parents/main caretakers or the mentor more in depth when the participant initiated the conversation about suicide, self-injury, sexual or physical abuse, or eating disorders. Girls will have to provide written informed consent for this part of the training separately before the PTT will start. The training sessions will be continued after this procedure.

All reported difficulties and support provided will be logged in the research team logbook. This part of the intervention protocol will be discussed and agreed with the headteachers/teacher-mentors of the participating dyads and will be explained in the participant information sheets for parents and girls.

Using the app

Because the App yourself Happy app will collect high-risk personal (health) data in order to monitor the program fidelity and to supervise the trainers (i.e., this process will be the responsibility of the PI), the Chief Privacy Officer of the Rotterdam University of Applied Sciences and the Data Steward conducted a Data Protection Impact Assessment (DPIA), which was finalized in December 2022. Rotterdam University of Applied Sciences and YipYip (i.e., the company who is leading on the technical development and design of the App yourself Happy app) have conducted a “Data Verwerkersovereenkomst.” In sum: all risks have been analyzed and this process resulted in several mitigating measures. The only identified residual risk with moderate risk status concerns the following: “Appropriate agreements have been made with YipYip and the VU about the processing of personal data recorded in a processing agreement. YipYip has an ISO/IEC27001:2017 and NEN 7510–1:2017 certification that are both valid until April 2025. YipYip uses the ‘subverwerker’ Google Cloud Platform. The company is in the United States, but data storage takes place in the Netherlands. YipYip has indicated to have appropriate agreements with Google and is Google ISO 27001 and NEN 7510 certified. Any residual risk at American cloud providers cannot be ruled out.”

## Data management

### Sharing during research

In order to facilitate co-operative research over long-distance and only if necessary, research data including personal data may be shared among the PI and all researchers (including the data manager). Data linked to published papers will be made openly available minus any data that can be considered personal data (e.g., video data). For the purpose of sharing pseudonymized research data over distance, researchers will use ResearchDrive or the Dutch cloud service SURFdrive. SURFdrive is designed specifically for higher education and research purposes and offers researchers and staff an easy and safe way to share and synchronize files within a secure community cloud with ample storage capacity. All SURFdrive information security protocols meet high standards. The Dutch Legal Framework for Cloud Services serves as a guideline for all service-related agreements. SURFdrive complies with Dutch and European privacy legislation. In addition, access to SURFdrive is password protected and designated folders can be password protected. Communication to and from SURFdrive is encrypted. If shared via SURFdrive, files that contain personal data are placed in designated and password-protected folders. In addition, such shared files will be encrypted. Keys to encrypted shared files are held by the PI and secondary researchers.

### Sharing after research

When the study is completed, the underlying research data may be shared with third parties for the purposes of reproduction, reuse, or assessment of scientific integrity. The sharing of research data connected to a publication is subjected to contractual obligations with the publisher. Data linked to published papers will be made openly available minus any data that can be considered personal data (e.g., video data). The sharing of research data and codebook with third parties will be carried out with the use of DataverseNL. Files that contain personal data will not be stored on DataverseNL unless they are anonymized and/or pseudonymized. Files that contain personal data will be stored in YODA, an online archive for storing sensitive information/data with a persistent identifier. Files stored in YODA will not be shared with third parties, unless the purpose of reproduction, reuse, or assessment of scientific integrity justifies otherwise. In that event such files may be released subject to identification of the requestor accompanied by a purpose statement and approval by the competent authority of the Vrije Universiteit Amsterdam.

### Dissemination policy

Research findings of the HFPM cRCT will be presented in open access high-quality peer review journals and via magazines for teacher- and health care professionals and through presentations at variety of academic conferences. Furthermore, the research findings will be presented to participants (i.e., girls, parents/main caretakers and schools) via management letters and the study website: www.happyfriends.app..

## Discussion

The proposed study aims to provide insight into the short-term (T1, T2, T3 and T4) and long-term effects (T5) of the HFPM secondary school-edition prevention program on girls’ co-rumination, internalizing problems, wellbeing, and social-emotional behavioral functioning.

The HFPM program is school-based mindfulness and psychoeducation blended prevention program aimed at girls aged 13–15 years within secondary schools. This program was developed between 2020 and 2023 using the Intervention Mapping Approach for planning health promotion programs. The program comprises of 14 guided, weekly online lessons with mindfulness practices and psychoeducation, guiding the dyadic use of the application App yourself Happy. The goal of this program is to train 160 Dutch (80 dyads) high-risk girls between ages 13 and 15 to shift dyadic maladaptive ER patterns to more adaptive ER strategies within their dyadic interactions, while continuing to reap the benefits of their close, intimate friendships and exploring healthy, new alternatives for excessive co-rumination.

The objective of the HFPM cRCT is to evaluate the effectiveness of the prevention program HFPM, delivered by experienced mindfulness health professionals, compared with CAU and to unveil mechanisms of change. Self-reported risk for (early onset) depression and anxiety and self-reported and observed co-rumination will be the primary outcomes. Secondary outcomes will be self- and friend-reported friendship quality, self-reported positive and negative affect, self-reported interpersonal reactivity to personal distress, self-reported interpersonal responses to positive affect, self-reported anhedonic symptoms, self-reported mastery, and parent-reported health care use.

If the trial demonstrates that this prevention program is effective, secondary schools will be able to prevent excessive co-rumination in young adolescent girls and to improve girls’ short- and long-term mental health outcomes and friendship quality. The trial will also contribute to our understanding for whom, and under which circumstances, the prevention program is most effective, so schools will be able to match the intervention working mechanisms with the specific needs of subgroups of girls within schools. This knowledge will result in continuous improvements in the development of the train-the-trainer program, including training and supervision of HFPM trainers in order to maximize implementation quality and fidelity.

### Supplementary Information

**Additional file 1: Figure S1.** Standard Protocol Items: Recommendations for Intervention Trials (SPIRIT) diagram detailing trial activities and measures and their timing.

**Additional file 2: Figure S2.** Multi-level power analyses: power versus number of clusters.

**Additional file 3: Table S1.** Specified duration: girls.

**Additional file 4: Table S2.** Specified duration: parents/caretakers.

**Additional file 5: Table S3.** Specified duration: trainers.

**Additional file 6: Figure S3.** Simplified visual representation of a multi-level, duals process latent growth model with a predictor (e.g., intervention condition), mediator (e.g., co-rumination) and outcome (e.g., internalizing problems). Ovals represent latent variables; squares represent observed variables. Double headed arrows represent (residual error) correlations, single headed arrows represent regression paths. Paths of interest are in bold.

**Additional file 7: Figure S4.** Simplified visual representation of a multilevel autoregressive crossed-lagged model with a predictor (i.e., intervention condition), mediator (i.e., co-rumination) and two correlated outcome variables (i.e., internalizing problems and negative affect). Ovals represent latent variables; squares represent observed variables. Double headed arrows represent (residual error) correlations, single headed arrows represent regression paths. Paths of interest are in bold.

**Additional file 8. **Statistical Analyses Plan.

## Data Availability

The datasets generated and/or analyzed during the current study will be stored in a publicly available repository. The repository that will be used for archiving is DataverseNL (https://dataverse.nl/dataverse/vuamsterdam) using CC BY-NC or a comparable license. Data that will be shared will be raw data, cleaned data, syntaxes, transcripts, and logbooks. This data will be accompanied by a codebook which can be referred to interpret the data. The data will be archived after the project is finished and will be retained for 10 years after the final publication. Where possible, data will be openly shared, but since the data concerns human subjects, it is likely an approved data request form will be required before data is issued (encrypted files will be sent via SurfDrive or similar secure transfer methods). Data issued will not include personal data unless it is absolutely required. Either way, steps will be taken to ensure the data is recoded (age instead of birthdate, no open questions, etc.) or anonymized.

## Abbreviations

APIM: Actor-Partner Interdependence Model
CAU: Care-as-usual
CCMO: Centrale Commissie Mensgebonden Onderzoek
CFI: Comparative Fit Index
CHIME-A: Comprehensive Inventory of Mindfulness Experiences-Adolescents
CoDEQ: Co-Dampening and Co-Enhancing Questionnaire
cRCT: Cluster Randomized Controlled Trial
DERS: Difficulties in Emotion Regulation
DPIA: Data Protection Impact Assessment
DTSA: Discrete-time survival analysis
ER: Emotion regulation
GAD: Generalized Anxiety Disorder
GCP: Good Clinical Practice
HFPM: Happy Friends, Positive Minds
IRI-PD: Interpersonal Reactivity Index for Personal Distress
ISCED: International Standard Classification of Education
Koers VO: Koers Voortgezet Onderwijs
LASS: Leuven Anhedonia Self-Report Scale
LCA: Latent Class Analysis
LTA: Latent Transition Analysis
LGM: Latent Grow Curve Model
MBI:TAC: Mindfulness-Based Interventions – Teaching Assessment Criteria
MLR: Maximum Likelihood estimation with Robust standard errors
MYRIAD: My Resilience in Adolescence
NRI: Network of Relationships Inventory
PANAS-C: Positive and Negative Affect Schedule for Children
PATS: Problem Anxiety Task Scale
PI: Principal Investigator
PMS: Pearling Mastery Scale
PTT: Problem Talk Task
RCADS: Revised Children’s Anxiety and Depression Scale
RMSEA: Root Mean Square Error of Approximation
RUAS: Rotterdam University of Applied Sciences
SCM: Self-Control Measure
SRMR: Standardized Root Mean Square Residual
SD: Standard deviation
SES: Socioeconomic status
SPIRIT: Standard Protocol Items: Recommendations for Intervention Trials
VU Amsterdam: Vrije Universiteit Amsterdam
WLSMV: Weighted least squares means and variance adjusted
WMO: The Dutch Medical Research Involving Human Subjects Act
