# Supplementary material for: Effectiveness of a blended school-based mindfulness program for the prevention of co-rumination and internalizing problems in Dutch secondary school girls: a cluster randomized controlled trial
Source: Trials. 2024 Jan 12;25:40. doi: 10.1186/s13063-023-07885-x (PMC10785508; doi:10.1186/s13063-023-07885-x)
Supplement: Supplementary file 1 — Additional file 1: Figure S1. Standard Protocol Items: Recommendations for Intervention Trials (SPIRIT) diagram detailing trial activities and measures and their timing. [file 13063_2023_7885_MOESM1_ESM.docx]

Figure 1. Standard Protocol Items: Recommendations for Intervention Trials (SPIRIT) diagram detailing trial activities and measures and their timing

|  | **Enrolment for both cohorts** | | **Alloca-tion for both cohorts** | **Post-allocation for both cohorts** | | | | | | | | | |
| --- | --- | --- | --- | --- | --- | --- | --- | --- | --- | --- | --- | --- | --- |
| **TIMEPOINT**** | ***-t_1_*** | **t0** | **Alloca-tion** | **Training** | ***Intervention*** | ***t_1_*** | ***Intervention*** | ***t_2_*** | ***Intervention*** | ***t_3_*** | ***Intervention*** | ***t_4_*** | ***t_5_*** |
| **Months relative to pre-intervention (t1) assessment** | **-12 to -2** | **-2** | **-2** | **-2** | **-1** | **0** | **1** | **2** | **3-4** | **5** | **6** | **7** | **19** |
| **ENROLMENT:** |  | | | | | | | | | | | | |
| **Headteacher consent** | X |  |  |  |  |  |  |  |  |  |  |  |  |
| **Parental consent** |  | X |  |  |  |  |  |  |  |  |  |  |  |
| **Girls’ consent** |  | X |  |  |  |  |  |  |  |  |  |  |  |
| **Screening** |  | X |  |  |  |  |  |  |  |  |  |  |  |
| **Randomisation** |  |  | X |  |  |  |  |  |  |  |  |  |  |
| **INTERVENTIONS:** |  | | | | | | | | | | | | |
| **Happy Friends, Positive Minds prevention program** |  |  |  |  | X |  | X |  | X |  | X |  |  |
| **Care as Usual (CAU)** |  |  |  |  | X |  | X |  | X |  | X |  |  |
| **GIRLS’ SELF REPORT:** |  | | | | | | | | | | | | |
| **CRQ-short** |  | X |  |  |  | X |  | X |  | X |  | X | X |
| **CDI-2** |  | X |  |  |  | X |  | X |  | X |  | X | X |
| **RCADS** |  | X |  |  |  | X |  | X |  | X |  | X | X |
| **PATS** |  | X |  |  |  | X |  | X |  | X |  | X | X |
| **PANAS-C** |  | X |  |  |  | X |  | X |  | X |  | X | X |
| **CoDEQ** |  | X |  |  |  | X |  | X |  | X |  | X | X |
| **NRI** |  | X |  |  |  | X |  | X |  | X |  | X | X |
| **IRI-PD** |  | X |  |  |  | X |  | X |  | X |  | X | X |
| **PMS** |  | X |  |  |  | X |  | X |  | X |  | X | X |
| **SCM** |  | X |  |  |  | X |  | X |  | X |  | X | X |
| **CHIME-A** |  | X |  |  |  | X |  | X |  | X |  | X | X |
| **DERS** |  | X |  |  |  | X |  | X |  | X |  | X | X |
| **Observations of co-rumination** |  | X |  |  |  |  |  |  |  |  |  | X |  |
| **Participant responsiveness (intervention group)** |  |  |  |  |  | X |  | X |  | X |  | X |  |
| **Participant practice outside training sessions (intervention group)** |  |  |  |  |  | X |  | X |  | X |  | X |  |
| **PARENTAL ASSESSMENTS OF GIRLS:** |  | | | | | | | | | | | | |
| **Health care use** |  | X |  |  |  |  |  |  |  |  |  | X |  |
| **Socio-economic status** |  | X |  |  |  |  |  |  |  |  |  |  |  |
| **Ethnicity** |  | X |  |  |  |  |  |  |  |  |  |  |  |
| **Treatment contamination (only control group)** |  | X |  |  |  |  |  | X |  |  |  | X |  |

| **TRAINER ASSESSMENTS:** |  | | | | | | | | | | | | |
| --- | --- | --- | --- | --- | --- | --- | --- | --- | --- | --- | --- | --- | --- |
| **Program dosage** |  |  |  |  |  | X |  | X |  | X |  | X |  |
| **Program fidelity** |  |  |  |  |  | X |  | X |  | X |  | X |  |
