# Supplementary figures and images for: Effectiveness of a blended school-based mindfulness program for the prevention of co-rumination and internalizing problems in Dutch secondary school girls: a cluster randomized controlled trial
Source: Trials. 2024 Jan 12;25:40. doi: 10.1186/s13063-023-07885-x (PMC10785508; doi:10.1186/s13063-023-07885-x)

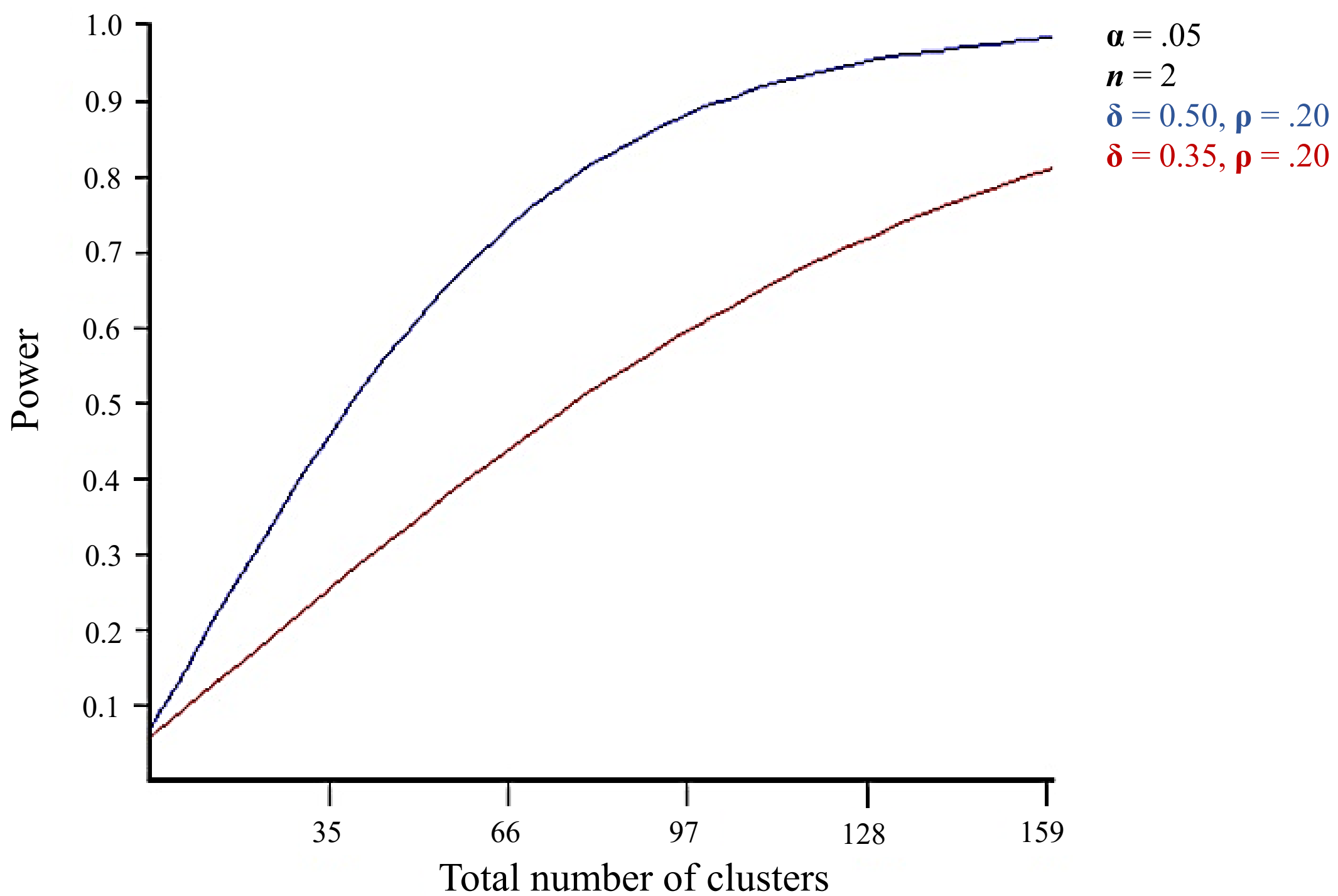

Supplement: Supplementary file 2 — Additional file 2: Figure S2. Multi-level power analyses: power versus number of clusters. [file 13063_2023_7885_MOESM2_ESM.pdf]
