# Supplementary material for: Effectiveness of a blended school-based mindfulness program for the prevention of co-rumination and internalizing problems in Dutch secondary school girls: a cluster randomized controlled trial
Source: Trials. 2024 Jan 12;25:40. doi: 10.1186/s13063-023-07885-x (PMC10785508; doi:10.1186/s13063-023-07885-x)
Supplement: Supplementary file 3 — Additional file 3: Table S1. Specified duration: girls. [file 13063_2023_7885_MOESM3_ESM.docx]

**Table 1. Specified duration: girls**

| **Questionnaire** | **Duration (min)** |
| --- | --- |
| **Screening** | |
| Co-rumination Questionnaire – CRQ short-5 | 1.25 |
| Total | 1.25 |
| **T0 t/m T4 and follow-up primary measures** | |
| Co-rumination Questionnaire – CRQ short –9 | 2.0 |
| CDI-2 | 10 |
| RCADS (Subscale Generalized Anxiety Disorder) | 1.5 |
| PATS | 5 |
| PANAS-C | 5 |
| CoDEQ | 5 |
| NRI | 10 |
| Total | 38.5 |
| **T0 t/m T4 secondary measures** | |
| IRI-PD | 5 |
| LASS | 3 |
| Mastery Scale | 5 |
| Total | 13 |
| **T0 t/m T4 and follow-up : moderator** | |
| Self-Control Measure | 2 |
| Total | 2 |
| **T0 t/m T4 and follow-up : mediators** | |
| CHIME-A | 10 |
| DERS | 10 |
| Total | 20 |
| **T1 t/m T4 implementation variables* only for girls in the intervention condition** | |
| Participant responsiveness | 28 |
| Practice outside of the training session | 3.5 |
| Total | 31.5 |
|  |  |
|  |  |
| Total Screening (min) | 1.25 |
| Total T0 t/m T4 (min p. assessment) | 73.5 |
| Total follow-up measures (min) | 60.5 |
| Total observations (T0 and T4) | 40 |
| Total implementation measures. only for girls in the intervention condition (min) | 31.5 |

**Table 2. Specified duration: parents/caretakers**

| **Questionnaires** | **Duration (min)** |
| --- | --- |
| **T0** | |
| Descriptives (SES and Ethnicity) | 3 |
| Total | 3 |
|  |  |
| **T0 en T4** | |
| Parental health care use of their child/family | 3 |
| Total | 3 |
|  |  |
| **T0. T2 en T4 : Implementation variables** | |
| Treatment contamination | 1 |
| Total | 1 |
|  |  |
| Total T0 | 7 |
| Total T2 | 1 |
| Total T4 | 4 |
