# Supplementary material for: Effectiveness of a blended school-based mindfulness program for the prevention of co-rumination and internalizing problems in Dutch secondary school girls: a cluster randomized controlled trial
Source: Trials. 2024 Jan 12;25:40. doi: 10.1186/s13063-023-07885-x (PMC10785508; doi:10.1186/s13063-023-07885-x)
Supplement: Supplementary file 4 — Additional file 4: Table S2. Specified duration: parents/caretakers. [file 13063_2023_7885_MOESM4_ESM.docx]

**Table 2. Specified duration: parents/caretakers**

| **Questionnaires** | **Duration (min)** |
| --- | --- |
| **T0** | |
| Descriptives (SES and Ethnicity) | 3 |
| Total | 3 |
|  |  |
| **T0 en T4** | |
| Parental health care use of their child/family | 3 |
| Total | 3 |
|  |  |
| **T0. T2 en T4 : Implementation variables** | |
| Treatment contamination | 1 |
| Total | 1 |
|  |  |
| Total T0 | 7 |
| Total T2 | 1 |
| Total T4 | 4 |
