# Supplementary material for: Effectiveness of a blended school-based mindfulness program for the prevention of co-rumination and internalizing problems in Dutch secondary school girls: a cluster randomized controlled trial
Source: Trials. 2024 Jan 12;25:40. doi: 10.1186/s13063-023-07885-x (PMC10785508; doi:10.1186/s13063-023-07885-x)
Supplement: Supplementary file 5 — Additional file 5: Table S3. Specified duration: trainers. [file 13063_2023_7885_MOESM5_ESM.docx]

**Table 3. Specified duration: trainers**

| **Questionnaires** | **Duration (min)** |
| --- | --- |
| **T1. T2. T3 and T4: Implementation variables** | |
| Program dosage for each girl | 70 |
| Program fidelity for each girl | 28 |
| Program quality: Mindfulness Based Interventions (MBI:TAC) | 1 |
| Total | 99 |
|  |  |
| **Total for each participating girl*** | 99 |

***Total amount of min. depends on the *n* of girls a trainer supports with the online training sessions**
