# Supplementary material for: Effectiveness of a blended school-based mindfulness program for the prevention of co-rumination and internalizing problems in Dutch secondary school girls: a cluster randomized controlled trial
Source: Trials. 2024 Jan 12;25:40. doi: 10.1186/s13063-023-07885-x (PMC10785508; doi:10.1186/s13063-023-07885-x)
Supplement: Supplementary file 8 — Additional file 8. Statistical Analyses Plan. [file 13063_2023_7885_MOESM8_ESM.docx]

**SUPPLEMENTARY MATERIAL**

**STATISTICAL ANALYSES PLAN**

This SAP is consistent with the principal features of the statistical methods described in the protocol *“Effectiveness of a blended school-based mindfulness program for the prevention of co-rumination and internalizing problems in Dutch secondary school girls: a Cluster Randomized Controlled Trial”*

**Statistical Methods for Primary Outcomes**

***Hypothesis 1 “Girls in the intervention group will have a greater reduction in co-rumination about distress and difficult emotions and feelings, (and thereby) internalizing symptoms and negative affect during the intervention period, immediately after the intervention period and after one-year follow-up, relative to girls in the control condition”*** will be tested via a) a multi-level parallel process, dual latent growth curve model (with co-rumination measured via self-report) and b) a multi-level autoregressive cross-lagged model (with co-rumination measured via observation).

For analysis a) with co-rumination measured via self-report (T0 to one-year follow up T5), we will use multi-level parallel process, dual latent growth curve (LGM) models, with intervention arm as the exogenous predictor variable, growth rate of co-rumination as the mediating variable and growth rate of internalizing symptoms and negative affect as (correlated) outcome variables. The mediator and outcome variables will be modelled on both the individual (within) level and on the dyadic (between) level. This way, the between-level intervention effects will be adjusted for variation between individuals in co-rumination, internalizing symptoms, and negative affect on the within-level. Note that we have no a priori hypotheses regarding the nature of the growth patterns of the mediator and outcome variables and that they may be best captured by either a linear or a non-linear slope. We will adjust the analyses accordingly. For a (simplified) visual representation of this model, please see Figure 1. For a more technical description of this model, we refer to the multilevel (mediation) SEM framework described by Preacher and colleagues (1, 2).

For analysis b) with co-rumination measured via observations (T0 and T4), we will use a multi-level autoregressive crossed-lagged model, with intervention arm as the exogenous predictor variable, (relative change from T0 to T4 in) co-rumination as the mediating variable and (relative change from T0 to T4 in) internalizing symptoms and negative affect as (correlated) outcome variables. De mediator and outcome variables will be modelled on both the individual (within) level and on the dyadic (between) level. This way, the between-level intervention effects will be adjusted for variation between individuals in co-rumination, internalizing symptoms, and negative affect on the within-level. For a (simplified) visual representation of this model, please see Figure 2. We refer to Preacher and colleagues for a more technical description of this model (1, 2).

**INSERT ADDITIONAL FILE 6 =**

| **Figure** **1**. Simplified visual representation of a multilevel, duals process latent growth model with a predictor (e.g., intervention condition), mediator (e.g., co-rumination) and outcome (e.g., internalizing problems). Ovals represent latent variables; squares represent observed variables. Double headed arrows represent (residual error) correlations, single headed arrows represent regression paths. Paths of interest are in bold. |
| --- |

**INSERT ADDITIONAL FILE 7 =**

| **Figure 2**. Simplified visual representation of a multilevel autoregressive crossed-lagged model with a predictor (i.e., intervention condition), mediator (i.e., co-rumination) and two correlated outcome variables (i.e., internalizing problems and negative affect). Ovals represent latent variables; squares represent observed variables. Double headed arrows represent (residual error) correlations, single headed arrows represent regression paths. Paths of interest are in bold. |
| --- |

**Statistical methods for secondary outcomes**

Hypothesis 2 **“Girls in the intervention group will have a a) later onset of depressive symptoms or a later onset of depressive disorder and b) less dyadic depression contagion during the intervention period, immediately after the intervention period and after one-year follow-up, relative to girls in the control condition”** will be tested using discrete-time survival analysis (3) and via actor-partner interdependence modeling (4), respectively.

Discrete-time survival analysis (DTSA), also known as “event history analysis” or “time-to-event analysis” will be used to investigate whether girls in the intervention group have a later onset of depression symptoms or disorder compared to girls in the control arm. DTSA enables to study the probability, or hazard, of experiencing a non-repeatable event, such as the onset of depressive symptoms and the onset of depression disorder. This type of analysis considers the timing as well as the occurrence of the first time an event (here: experiencing depressive symptoms for the first time or receiving a clinical diagnosis of depression for the first time), and thus allows for examining the longitudinal progression of the likelihood that the onset of the event occurs within the measurement intervals (T0 – one-year follow up). DTSA models use right-censored data and therefore properly account for the fact that many adolescents will not experience the event during the measurement period. That is, although we will include a high-risk group of participants, it is likely that many will not receive a depression diagnosis during the study period. Furthermore, DTSA can be combined with exogeneous predictors (here: intervention condition) to investigate whether the time-invariant predictor intervention arm is associated with the probability of an event (diagnosis/symptoms) occurring (3). Note that this research question focuses on individual rather than dyadic outcomes. To adjust for individuals clustered within dyads, we will adjust the standard errors using a sandwich estimator (5).

Prospective change, a Actor-Partner Interdependence Model (APIM) will be used to investigate whether dyads in the intervention condition will experience less dyadic depression contagion compared to dyads in the control arm from T0 – one-year after follow-up. APIM allows to test the effects of each girl’s predictor variables (here: depressive symptoms) on her own outcomes (actor effects) and on her friend’s outcomes (partner effects), while controlling for similarities between dyad-members. If the actor effect is non-significant, this indicates stability in depression symptoms. If the actor effect is significant, this indicates contagion between girls in the dyad. We will use multi-group comparisons to test whether contagion effects are less strong in the intervention arm compared to the control arm. Specifically, a model is which the actor and partner effects will be estimated freely for the intervention and control arm (unconstrained model) versus a model where the actor and partner effects will be constrained to be equal will be specified. If constraining the parameters to be equal results in poorer model fit, this indicates that effects are not similar in the two conditions.

Hypothesis 3 **“Girls in the intervention group will demonstrate a) less anxiety problem talk and will have a b) later onset of anxiety symptoms or a later onset of anxiety disorder and c) less dyadic anxiety contagion during the intervention period, immediately after the intervention period and after one-year follow-up, relative to girls in the control condition”** will be tested using a multilevel LGM, DTSAs and multi-group APIM, respectively.

A multi-level LGM with intervention arm as the exogenous predictor variable and growth rate of anxiety problem talk as the outcome variable will be used to test whether girls in the intervention arm demonstrate less anxiety problem talk compared to girls in the control arm. Anxiety problem talk will be modelled on both the individual (within) level and on the dyadic (between) level. This way, the between-level intervention effects will be adjusted for variation between individuals in anxiety problem talk on the within-level. Note that we have no a priori hypotheses regarding the nature of the growth patterns of anxiety problem talk and that it may be best captured by either a linear or a non-linear slope. The analysis will be adjusted accordingly.

DTSAs of anxiety symptom onset and anxiety disorder onset with intervention condition as an exogenous predictor will be used to test whether girls in the intervention group have a later onset of anxiety symptoms or disorder compared to girls in the control arm. For a more detailed description of this model, please see the model description in hypothesis 2a.

Multi-group (i.e., intervention versus control condition) APIM will be used to investigate whether dyads in the intervention condition will experience less anxiety contagion compared to dyads in the control arm from T0 to one-year after follow up. For a more detailed description of this model, please see the model description in hypothesis 2b.

Hypothesis 4 **“Girls in the intervention group will experience better friendship quality, higher levels of positive affect and higher levels of interpersonal responses to positive affect of the dyad friend, during the intervention period, immediately after the intervention period and after one-year follow-up, relative to girls in the control condition”** will be tested using multi-level LGMs for each outcome, respectively.

Three multi-level LGMs with intervention arm as the exogenous predictor variable and growth rate of friendship quality, positive affect and interpersonal responses to positive affect as the (potentially correlated) outcome variables, will be used to test whether girls in the intervention arm experience more improvements in friendship quality, a faster growth in positive affect and faster growth levels of interpersonal responses to positive affect, compared to girls in the control arm. The outcome variables will be modelled on both the individual (within) level and on the dyadic (between) level. This way, the between-level intervention effects will be adjusted for variation between individuals in the outcome measures on the within-level. Note that we have no a priori hypotheses regarding the nature of the growth patterns of the outcome measures and that it may best captured by either a linear or a non-linear slope. The analysis will be adjusted accordingly.

Hypothesis 5 ***“The hypothesized intervention effects on co-rumination will be mediated by the development of mindfulness skills, emotion regulation skills and problem-solving skills during the intervention period, immediately after the intervention period and after one-year follow-up”*** will be tested using a) a multi-level parallel process LGMs (for self-reported co-rumination) and b) a multi-level autoregressive cross-lagged model (with co-rumination measured via observation).

For the model where co-rumination is measured via self-report, a multi-level (LGM) model, with intervention arm as the exogenous predictor variable, growth rate of mindfulness skills, emotion regulation skills and problem-solving skills as the mediating variables and growth rate of co-rumination as the outcome variable. The mediator and outcome variables will be modelled on both the individual (within) level and on the dyadic (between) level. This way, the between-level intervention effects will be adjusted for variation between individuals in co-rumination, internalizing symptoms and negative affect on the within-level. Note that we have no a priori hypotheses regarding the nature of the growth patterns of the mediator and outcome variables and that they may be best captured by either a linear or a non-linear slope. The analysis will be adjusted accordingly.

For the model with co-rumination measured via observations (T0 and T4), we will use a multi-level autoregressive crossed-lagged model, with intervention arm as the exogenous predictor variable, (relative change from T0 to T4 in) mindfulness skills, emotion regulation skills and problem-solving skills as the (correlated) mediating variables and (relative change from T0 to T4 in) co-rumination as the outcome variable. De mediator and outcome variables will be modelled on both the individual (within) level and on the dyadic (between) level. This way, the between-level intervention effects will be adjusted for variation between individuals in the mediating variables and the outcome variable on the within-level.

Hypothesis 6 ***“The hypothesized intervention effects on co-rumination will be moderated by self-control: girls with more developed self-control skills will demonstrate greater intervention effects immediately after the intervention period and after one-year follow-up, relative to girls in the control condition”*** will be tested a) a multi-level parallel process moderation LGM (for self-reported co-rumination) and b) a multi-level autoregressive cross-lagged model (with co-rumination measured via observation).

The hypothesis that the intervention effects on self-reported co-rumination development will be moderated by development in self-control will be tested using a multi-level LGM. In this model, intervention arm is an exogenous predictor of the (between-level) growth curve of outcome variable co-rumination and growth in self-report from T0 to one-year after follow-up is the moderator. Thus, interactions between the slope of the moderator self-control and the slope of the outcome variable co-rumination will be added to the main effect model to test whether the path between intervention condition (intervention versus control group) and self-reported co-rumination development is moderated by growth in self-control. Co-rumination and self-control will be modelled on both the individual (within) level and on the dyadic (between) level. This way, the between-level intervention effects will be adjusted for variation between individuals in the outcome and moderating variables on the within-level. Note that we have no a priori hypotheses regarding the nature of the growth patterns of the outcome and moderating variables and that it may best captured by either a linear or a non-linear slope. Analyses will be adjusted accordingly. See also Preacher and colleagues’ on multilevel SEM for assessing moderation within and across levels of analysis (6).

For analysis b) with co-rumination measured via observations (T0 and T4), we will use a multi-level autoregressive crossed-lagged model, with intervention arm as the exogenous predictor variable, (relative change from T0 to T4 in) self-control as the moderating variable and (relative change from T0 to T4 in) co-rumination as the outcome variables. De mediator and outcome variables will be modelled on both the individual (within) level and on the dyadic (between) level. This way, the between-level intervention effects will be adjusted for variation between individuals in co-rumination, and self-control on the within-level.

Hypothesis 7 ***“Girls in the intervention group will experience less anhedonic symptoms, will experience greater feelings of mastery and will show less health care use, immediately after the intervention period and after one-year follow-up, relative to girls in the control condition”*** will be tested using multi-level LGMs for each outcome, respectively. Five multi-level LGMs with intervention arm as the exogenous predictor variable and growth rate of anhedonic symptoms, feelings of mastery, and health care use as the (potentially correlated) outcome variables, will be used to test whether girls in the intervention arm experience less anhedonic symptoms, greater feelings of mastery, and less health care use over time, compared to girls in the control arm. The outcome variables will be modelled on both the individual (within) level and on the dyadic (between) level. This way, the between-level intervention effects will be adjusted for variation between individuals in the outcome measures on the within-level. Please note that health care use will be modelled as a categorical LGM due to the nature of the data (dichotomous). Furthermore, note also that we have no a priori hypotheses regarding the nature of the growth patterns of the outcome measures and that it may best captured by either a linear or a non-linear slope. We will adjust the analysis accordingly.

Hypothesis 8 ***“Girls in the intervention group will demonstrate a change in subjects discussed: girls will demonstrate less problem talk about interpersonal problems and shorter periods of interpersonal problem talk immediately after the intervention period and after one-year follow-up, relative to girls in the control condition”*** will be tested using Latent Transition Analysis. Latent Transition Analysis (LTA) is an extension of Latent Class Analysis (LCA) in which the probabilities of transitions among behavior patterns (here: amount and period of interpersonal subjects discussed) over time are estimated. In an LTA, a LCA at each time point is estimated and additionally estimates the probability of transitioning from each class at one time point to all others at the next time point. In our case, classes might be class 1: girls who engage a lot in interpersonal problem talk and who spend a lot of time on interpersonal problem talk during the co-rumination observation and class 2: girls who don’t engage a lot in interpersonal problem talk and who don’t spend a lot of time on interpersonal problem talk during the co-rumination observation. Any other number of classes might also appear, e.g., there might be an intermediate class as well. Next, via LTA it can be tested whether girls transition from one class to another over time (i.e., between T0 and T4). Via multi-group testing we will investigate whether the probability of changing from class 1 to class 2 is higher for girls in the intervention arm compared to girls in the control arm.

Hypothesis 9 ***“The hypothesized intervention effects on (the onset) of depression and anxiety symptoms or disorders and depression contagion and anxiety contagion are differently related to changes in interpersonal components of the conversations between girls”*** will be tested via a joint LTA and DTSA model. First, classes of girls who do and who do not change in their amount and period of interpersonal subjects discussed will be identified via an LTA. Next, it will be investigated whether the resulting classes retrieved from the LTA (i.e., probability of class-membership for the identified transition classes) is a mediator between the intervention effect and the DTSA model of the onset of depression and anxiety symptoms or disorders. For specific details about the LTA and DTSA models, see the description in hypothesis 2 and hypothesis 8, respectively.

All analyses will be conducted in structural equation modeling program Mplus (v 8.7 or higher; [109]). Model fit will be determined using the comparative fit index (CFI, critical value ≥ .95), the root mean square error of approximation (RMSEA, critical value ≤ .06) and the standardized root mean square residual (SRMR, critical value <. 08; [110]). When appropriate, standard errors will be adjusted for clustering of dyads within schools using a sandwich estimator [107]. For hypotheses that include mediation analysis (hypothesis 1 & 5) we will use 10.000 bootstrap resamples with replacement and bias-corrected 95% confidence intervals to test these indirect effects [111]. When appropriate, differences in (indirect) pathways between the intervention and the control arm will be estimated using the DIFFtest option in Mplus (when using the WLSMV estimator) or the Satorra Bentler chi-square difference test [113]; when using the MLR estimator). Missing data during the follow-up period (e.g., due to unavailability during a certain measurement wave or due to drop-out) will be handled using Full Information Maximum Likelihood (FIML) estimation, in which the population parameters are estimated that would most likely produce the estimates from the sample data that is analyzed.
