# Supplementary material for: Effectiveness of a blended school-based mindfulness program for the prevention of co-rumination and internalizing problems in Dutch secondary school girls: a cluster randomized controlled trial
Source: Trials. 2024 Jan 12;25:40. doi: 10.1186/s13063-023-07885-x (PMC10785508; doi:10.1186/s13063-023-07885-x)
Supplement: Supplementary file 7 [file 13063_2023_7885_MOESM6_ESM.docx]

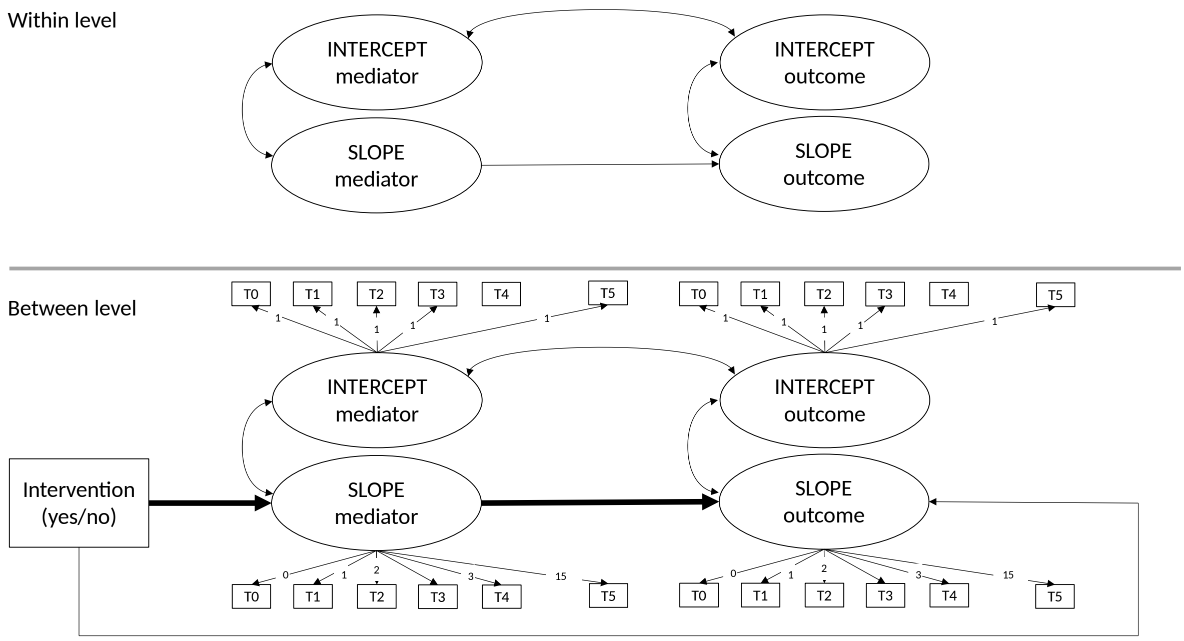

Within level
INTERCEPT mediator
INTERCEPT outcome
SLOPE mediator
SLOPE outcome
Between level
T0
T1
T2
T3
T4
T5
1
INTERCEPT mediator
INTERCEPT outcome
Intervention (yes/no)
SLOPE mediator
SLOPE outcome
0
1
2
3
15
