# Supplementary material for: Effectiveness of a blended school-based mindfulness program for the prevention of co-rumination and internalizing problems in Dutch secondary school girls: a cluster randomized controlled trial
Source: Trials. 2024 Jan 12;25:40. doi: 10.1186/s13063-023-07885-x (PMC10785508; doi:10.1186/s13063-023-07885-x)
Supplement: Supplementary file 8 [file 13063_2023_7885_MOESM7_ESM.docx]

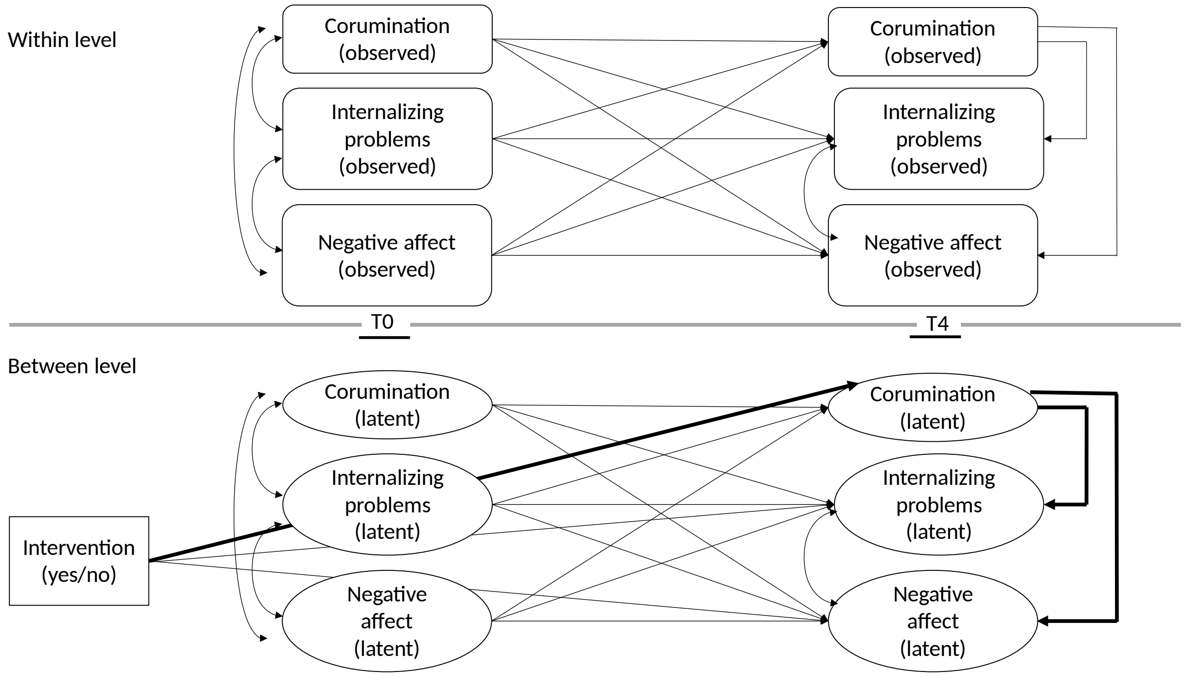
Within level
Corumination (observed)
Internalizing problems (observed)
Negative affect (observed)
Corumination (observed)
Internalizing problems (observed)
Negative affect (observed)
T0
T4
Between level
Corumination (latent)
Internalizing problems (latent)
Negative affect (latent)
Intervention (yes/no)
Corumination (latent)
Internalizing problems (latent)
Negative affect (latent)
